# Supramolecular Approach to Tuning the Photophysical Properties of Quadrupolar Squaraines

**DOI:** 10.3389/fchem.2021.800541

**Published:** 2022-01-05

**Authors:** Anna Kaczmarek-Kȩdziera, Borys Ośmiałowski, Piotr S. Żuchowski, Dariusz Kȩdziera

**Affiliations:** ^1^ Faculty of Chemistry, Nicolaus Copernicus University in Toruń, Toruń, Poland; ^2^ Institute of Physics, Nicolaus Copernicus University in Toruń, Toruń, Poland

**Keywords:** squaraine dye, photophysical properties, DFT calculations, interaction energy, SAPT energy decomposition, one-photon absorption, hydrogen bonding, non-additivity

## Abstract

In the present study, the influence of the hydrogen bonding for the one- and two-photon absorption of the prototypical squaraine dye is investigated with quantum chemistry tools. The central squaraine unit is bound by strong hydrogen bonds with 4-substituted N,N′-diphenylurea and, alternatively, N,N′-diphenylthiourea molecules, which affects to a high extend the properties of the squaraine electron accepting moiety, thus shifting its maximum absorption wavelength and enhancing the TPA cross section. The replacement of oxygen by sulfur atoms in the squaraine central ring, known to affect its photophysical behavior, is considered here as the way of modifying the strength and nature of the intermolecular contacts. Additionally, the influence of the oxygen-by-sulfur replacement is also considered in the N,N′-diphenylurea moiety, as the factor affecting the acidity of the N–H protons. The introduction of the sequence of the substituents of varying electron-donating or electron-withdrawing characters in the position 4 of N,N′-diphenyl(thio)urea subsystems allows to finely tune the hydrogen bonding with the central squaraine unit by further modification of the N–H bond characteristics. All of these structural modifications lead to the controlled adjustment of the electron density distribution, and thus, the properties affected such as transition moments and absorption intensity. *Ab initio* calculations provide strong support for this way of tailoring of one- or two-photon absorption due to the obtained strong hypsochromic shift of the maximum one-photon absorption wavelength observed particularly for thiosquaraine complexes and an increase in the TPA wavelength together with the increase in the TPA cross section. Moreover, the source of the strong modification of the thiosquaraine OPA in contrast to the pristine oxosquaraine upon N,N′-diphenyl(thio)urea substitution is determined. Furthermore, for the first time, the linear dependence of the non-additivity in the interaction energy on the Hammett substituent constant is reported. The stronger the electron-donating character of the substituent, the larger the three-body non-additive components and the larger their percentage to the total interaction energy.

## 1 Introduction

The growing interest in the applications of chromophores and fluorophores in the fields of biomedical techniques such as bioimaging or photodynamic therapy or in photovoltaic devices requires the rational design of the photoactive systems with respect to both their photophysical characteristics and solubility, stability, non-toxicity, and availability. Despite the numerous classes of molecules exhibiting the desired features at least in one of these fields (for instance, azobenzenes, merocyanines, coumarins, phthalocyanines, or dipyrromethene derivatives, just to mention a few), the continuous need of the precise and controlled tuning of their properties increases the number of contributions devoted to their modifications.

Squaraine dyes belong to the class of quadrupolar molecules of high interests in material chemistry, bioimaging, non-linear optics, or photonics. Their peculiar photooptical properties arise from their unique structure: an electron-deficient four-membered squaric acid ring (denoted further by A as acceptor) is placed in between two electron-rich donating groups (denoted by D as donors). This D–A–D structure results in a specific strong and sharp one-photon absorption (OPA) in the visible or near-IR region, exceptional brightness, and unique non-linear properties. However, squaraines are rarely used as probes or in biomedical applications due to their reported low solubility and lack of stability in biological media Ros-Lis et al. (2002); Ros-Lis et al. (2004); Karpenko et al. (2015). Additionally, the extended *π*-electron scaffold of squaraines promotes the stacking intermolecular interactions in polar solvents, causing the fluorescence quenching Arunkumar et al. (2007).

Numerous structural modifications of squaraine dyes have been investigated with respect to the particular features required for given applications. It is well-known that the proper introduction of the electron-withdrawing (EW) and electron-donating (ED) moieties to the molecular framework allows to finely tune both the maximum absorption wavelength and the TPA cross section, *σ*_TPA_. It has been demonstrated only recently that the terminal electron–withdrawing substituents inserted symmetrically in the indolenine squaraine dyes affect the transition dipole moments, the difference between static ground and excited state dipole moment, and absorption wavelength stronger than the electron-donating groups placed alike in the study by Barcenas et al. (2021). However, the maximum absorption wavelength in both cases (EW and ED) is shifted bathochromically and other properties considered by Barcenas et al. are also modified in the same direction for electrons being transferred by the substituent from or to the central squaric ring moiety. This can be perceived as a severe limitation of the tailoring strategies for the squaraine dyes in context of their desired features vital for further usage.

The applications of squaraines as photosensitizers in photodynamic therapy or in photon upconversion demands the efficient generation of stable triplet states. The enhancement of the intersystem crossing can be achieved by the introduction of heavy atoms. However, even only the oxygen-to-sulfur replacement in the squaric acid ring has been shown to increase the quantum yield for the triplet excited state, and thus it make squaraines the attractive triplet state photosensitizers with relatively weak modification of other photophysical properties, such as two-photon absorption Webster et al. (2010); Avirah et al. (2012); Peceli et al. (2013).

The presence of the D–A–D motif may lead to the efficient two-photon absorption (TPA) of squaraine systems. The simple symmetrical dibutylaniline squaraine dye (SQ) has been shown to exhibit the strong two-photon absorption with three bands: vibronic coupling band of the TPA cross section equal to 200 GM, second band at 850 nm with *σ* ^TPA^ = 2000 GM corresponding to the S_2_ excited state, and the third one at 700 nm with the cross section of 15,000 GM is governed by the excitation to the S_4_ state (Webster et al., 2010; Ferrer et al. (2019)). Squaraines of the extremely large values of *σ*_TPA_ have been obtained (for instance, 27 ,000 GM for heterocyclic pyrrole-substituted squaric acid ring) (Pawlicki et al., 2009). Among the popular techniques of optimization of the TPA properties of chromophores one should mention the extension of the *π*-electron–conjugated chain of the system, modification of the electron-donating and electron-accepting properties of the central and side moieties, or the conformational flexibility of the parts of the system. However the development of the whole molecule often aggravates the practical applications of the obtained system, for instance, significantly decreasing the solubility. For that reason, it is desired to design small molecules exhibiting the enhanced non-linear response.

**FIGURE 1:**
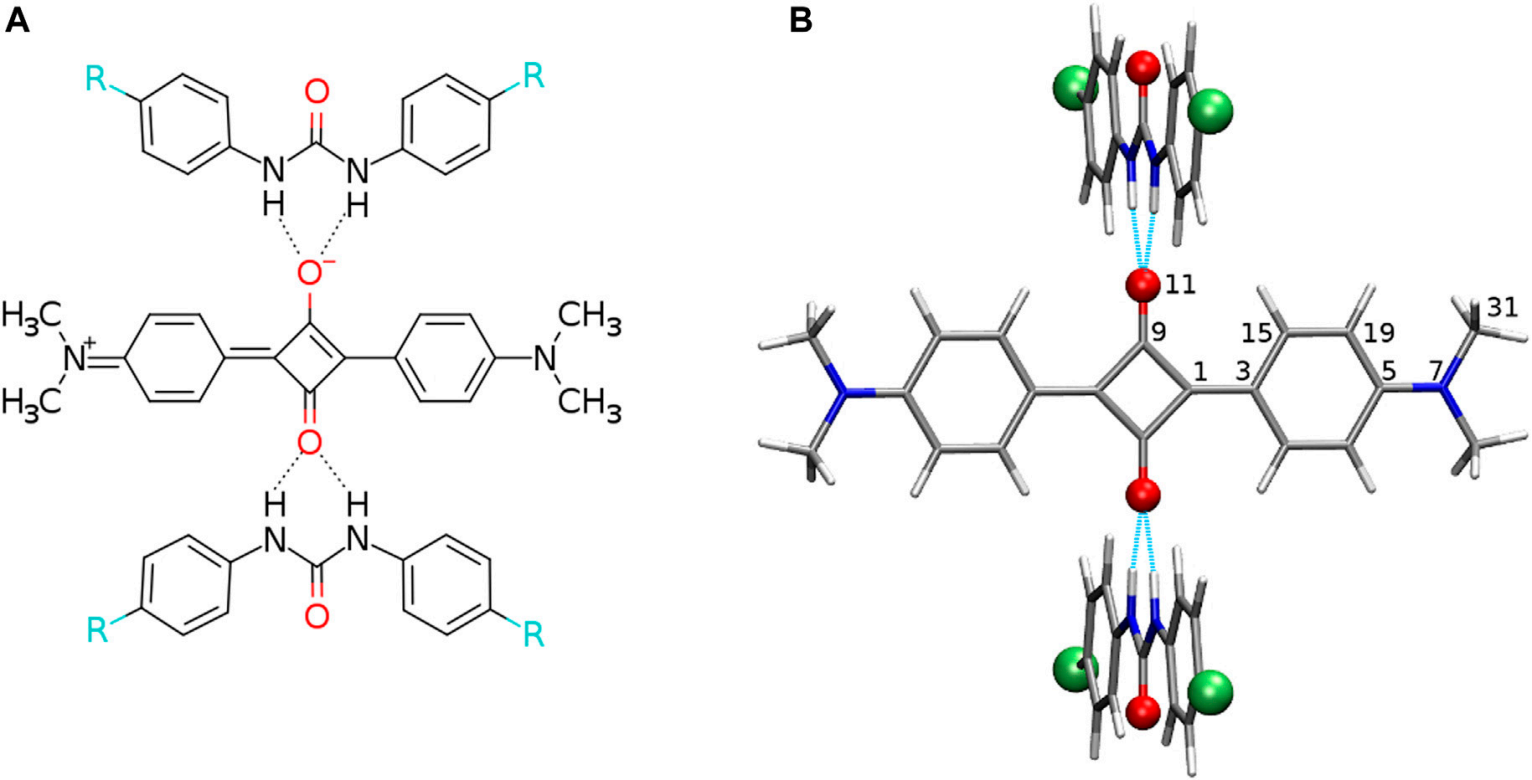
**(A)** Structural formula of the analyzed hydrogen-bonded squaraine complex with 2 N,N′-diphenylurea molecules (R denotes electron-donating or electron-accepting functional groups) and **(B)** exemplary optimized structure with the urea plane perpendicular to the squaraine plane (red balls placed for oxygen or sulfur atoms, green balls depict the protons substituted with electron-donating or electron-accepting functional groups, and intermolecular hydrogen bonds are shown in cyan dashed line). Atom numbering for the symmetrical and unique atoms is given.

It has been shown that the hydrogen bonding (HB)–governed aggregation can lead to the enhanced TPA (K. Liu and Y. Wang and Y. Tu and H. Å gren and Y. Liu (2007), Liu (2008)). Therefore, one could exploit the supramolecular architecture in order to improve the squaraine two-photon optical properties, at the same time aiming at the more flexible yet controllable variations of the dipole moments upon excitation or photophysical parameters. Among the systems widely investigated in the field of hydrogen-bonded supramolecular aggregation, the N,N′-diphenylurea (DPU) and their derivatives have occurred particularly interesting due to their easy crystallization and a well-defined crystal structure. This allows us to study the influence of the substitution effects on the structural and electronic properties of the aggregates. Since the DPU moiety possesses two strong hydrogen bond donors, it is expected to form stable and regular structures with a matching hydrogen bond acceptor. One can thus construct supramolecular systems built of the squaraine central moiety caught by the two urea derivatives forming the bifurcated hydrogen bonds to each of the oxygen atoms of the central four-membered squaraine ring, as presented schematically in Figure 1. The application of the N,N′-diphenylurea allows to finely tune the acidity of the N–H protons by the appropriate choice of the substituents in position 4 of the phenyl rings. Additionally, a similar sequence of the systems could be obtained by the utilization of the thiourea despite the urea system, since they are known to exhibit higher acidity of the N-H protons and are widely applied in organocatalysis.

The hydrogen bonding properties of numerous molecules can be influenced by substitution of the oxygen atom by sulfur. This was exemplified by numerous carbonyl and thiocarbonyl compounds (Hinchliffe, 1984; Platts et al., 1996; Allen et al., 1997; Rablen et al., 1998; Steiner, 2000; Krepps et al., 2001; Wennmohs et al., 2003; Valdés-Martínez et al., 2004; Jabłoński et al., 2006; Corpinot et al., 2017). The sulfur atom is known to be significantly weaker hydrogen bond acceptor than oxygen but also to exhibit the preference for larger deviation from 180° than in oxygen moieties (Platts et al., 1996). This arises from the fact, that opposite to the charge–charge interaction dominating in the H … O contacts, the hydrogen–sulfur attraction is stabilized by the charge–quadrupole interaction. Therefore, from the point of view of the photophysical properties of the systems investigated in the present study, the application of the sulfur derivatives of the squaraine dyes can be also vital. This modification is expected to change the order of the excited states of the squaraine moiety by introducing the low-lying *n* → π* transition exploiting the electron pair of sulfur. Hence, the reversed order of the *n* → π* and *π* → π* with respect to the original oxygen-containing squaraines allow for the stronger intersystem crossing according to the El Sayed rule (El-Sayed (1963)) and so further on, for instance, the enhancement of the singlet oxygen generation quantum yield necessary for the photodynamic therapy applications Peceli et al. (2013); Chetti et al. (2021). It has been shown that simple oxygen-by-sulfur substitution in both places in squaric ring in squaraines drastically modifies neither the one-photon nor the two-photon absorption of the dye. However, it occurs to be a convenient tool for the subtle tuning of the photooptical properties of squaraines and has got even more attention recently in the design of triplet–triplet annihilation systems for photon upconversion Peceli et al. (2013); Pristash et al. (2020); Chetti et al. (2021). The importance of this strategy for the rational design of fluorophores exhibiting bathochromic shift of the absorption and emission bands has been confirmed earlier also for different class of systems (Jedrzejewska et al., 2016).

Therefore, in the present study, the photophysical properties of the sequence of the supramolecular aggregates composed of the squaraine or thiosquaraine central moiety hydrogen bonded by the 2 N,N′-diphenylurea or 2 N,N′-diphenylthiourea (DPTU) derivatives are analyzed with the computational chemistry tools. The aim is to determine the structural modifications which allow tuning the one- or two-photon absorption and singlet–triplet energy gap in oxo- and thiosquaraines. The sequence of the electron-donating and electron-accepting substituents in the 4-position of the phenyl ring of the DPU and DPTU systems has been selected according to the Hammett electronic effects (Hansch et al., 1991). The systematic change of the substituent can be used to tune the intermolecular interactions but also may be used as a research tool to study the given system systematically (Ośmiałowski et al., 2013).

One need to underline that the prototypical dimethylaniline squaraine dye is challenging from the theoretical point of view due to the interplay of several competing effects: the charge transfer between the electron-accepting central squaric acid ring and the electron-donating side groups, the mild biradicaloid character, and the significant double excitations affecting the two-photon theoretical description (Prabhakar et al., 2005b; Prabhakar et al., 2010; Prabhakar et al., 2005a; Yesudas et al., 2006; Srinivas et al., 2007; Ferrer et al., 2019).

## 2 Methods

The complexes of squaraine (OSQ) and thiosquaraine (SSQ) with 2 N,N′-diphenylurea and N,N′-diphenylthiourea molecules have been investigated for the geometry frozen at the D_2*h*_ symmetry point group, as presented in Figure 1, in order to separate electronic effect from structural deformation. The modification of the acidity of the N–H protons of urea by the proper site substitution or O-by-S replacement is expected to affect both the N–H … O/N–H … S distances, electron density distribution *ρ*, and several other properties such as quadrupole moment, thus influencing intermolecular interaction energy and photophysics of the investigated chromophore. Therefore, the 4-substituted derivatives of urea and thiourea are analyzed. The sequence of the substituents is selected in order to ensure the wide range of electron-donating to electron-accepting properties: –NMe_2_, –NH_2_, –NHNH_2_, –OH, –NHOH, –CMe_3_, –Me, –H, –Cl, –CONH_2_, –CHO, –CCl_3_, –CF_3_, –COCl, –CN, and –NO_2_ (Hansch et al., 1991). The corresponding values of *σ*_*p*_ for these substituents are presented in Table 1. In order to establish the influence of the hydrogen bonds on the charge transfer inside the squaraine moiety, the artificially symmetrized perpendicular structures have been investigated (Figure 1). For these systems, $D_{2h}$ symmetry has been enforced and partial optimization has been performed in order to allow for the restricted geometry relaxation only. This arises from the fact that the extended *π*-electron aromatic scaffolds of squaraines prefer the dispersion interaction with the phenyl rings of the urea, when optimized freely. However, in order to limit the considerations to the hydrogen-bonded complexes, which could be observed in crystals, among other interactions, and affect the properties of the analyzed systems significantly, the free relaxation to the stacked architecture has been forbidden in the present study. The *ω*B97X-D functional has been chosen for its wide applicability and good performance for the molecular and aggregate structures (Chai and Head-Gordon, 2008a,b). The choice of the 6-31+G(d) basis set has been imposed by the presence of polarization and diffuse functions for heavy atoms accompanied by the moderate size of the basis set.

**TABLE 1:** Hammett constant values *σ*_*p*_ for the sequence of the analyzed substituents (Hansch et al., 1991).

	—	—	—	—	—	—	—	—
Substituent	–NMe_2_	–NH_2_	–NHNH_2_	–OH	–NHOH	–CMe_3_	–Me	–H
*σ*_*p*_	−0.83	−0.66	−0.55	−0.37	−0.34	−0.20	−0.17	0.00
Substituent	–Cl	–CONH_2_	–CHO	–CCl_3_	–CF_3_	–COCl	–CN	–NO_2_
*σ*_*p*_	0.23	0.36	0.42	0.46	0.54	0.61	0.66	0.78

Mutual interactions between the squaraine and urea derivatives have been investigated within the supermolecular approach. The counterpoise-corrected interaction energy $\Delta E_{\text{SM}}^{\text{CP}}\left(ABC\right)$ has been determined for the three-body system according to the site–site counterpoise procedure (Wells and Wilson, 1983; Richard et al., 2018) as follows:

$$\Delta E_{\text{SM}}^{\text{CP}}\left(ABC\right)=E_{\text{ABC}}\left(ABC\right)-E_{\text{ABC}}\left(A\right)-E_{\text{ABC}}\left(B\right)-E_{\text{ABC}}\left(C\right),$$ (1)

where A, B, and C denote one of the subsystems, respectively, while *E*_ABC_(*X*) stands for the energy of the subsystem X = A,B,C calculated in the basis set of the whole complex (basis set indicated in the subscript). The geometry of the subsystems was considered frozen at the complex geometry and no relaxation was allowed.

The non-additivity arising from the three-body effects is estimated as the difference between the total interaction energy in the trimer, $\Delta E_{\text{SM}}^{\text{CP}}\left(ABC\right)$ , and the sum of the interaction energies in dimers as follows (Chałasinski et al., 1991; Elrod and Saykally, 1994; Góra et al., 2011):

$$\Delta_{\text{non}-\text{add}}=\Delta E_{\text{SM}}^{\text{CP}}\left(ABC\right)-\left[\Delta E_{\text{SM}}^{\text{CP}}\left(AB\right)+\Delta E_{\text{SM}}^{\text{CP}}\left(AC\right)+\Delta E_{\text{SM}}^{\text{CP}}\left(BC\right)\right].$$ (2)

All the contributions here are calculated within the full trimer basis set (Řezáč et al., 2015) with *ω*B97X-D, MP2, and DLPNO-CCSD(T) approaches.

The energy of the hydrogen bonds in the investigated complexes is estimated on the basis of the theory of atoms in molecules according to Espinosa et al. (1998). The analysis of the partial charge distribution has been carried out with the natural population analysis (Foster and Weinhold, 1980; Reed et al., 1988) because of its small dependence on the basis set size, and the quality of the obtained data has been verified with Hirshfeld charges (Hirshfeld, 1977), presented in Supplementary Material for OSQ-DPU and SSQ-DPU complexes. The modification of the charge distribution upon excitation has been investigated with the Le Bahers indexes (Bahers et al., 2011) and Δ*σ* defined as the difference for the root-mean-square deviation of distribution for particle and hole is given in Supplementary Material.

Since the bis(N,N-dimethylamine) squaraine molecule represents the challenge for the computational methods due to the double excitation effects balanced with the charge transfer and moderate biradicaloid character, the literature recommendations have been applied for the computational methodology for the electronic excitations (Quartarolo et al., 2009; Alberto et al., 2014; Bassal et al., 2017; Ferrer et al., 2019). It has been proven that the conventional long-range–corrected functionals correctly predict the one-photon absorption spectrum of bis(N,N-dimethylamine) squaraine and despite strong overestimation of the energy of the states involved in two-photon transitions allow the correct assignment of the experimentally observed states. Therefore, the one-photon and two-photon absorption has been described within the Coulomb-attenuated B3LYP functional (Beerepoot et al., 2018). For comparison, OPA parameters were also determined with M06-2X and PBE0 functionals and are provided in Supplementary Material (Zhao and Truhlar, 2008; Jacquemin et al., 2012; Leang et al., 2012; Isegawa et al., 2012; Charaf-Eddin et al., 2013; Houari et al., 2014; Azarias et al., 2016).

According to the selection rules for OPA, the transitions in the centrosymmetric systems are possible only with the change of the symmetry of the state (from gerade to ungerade or from ungerade to gerade), in opposition to the TPA-allowed electric dipole transitions, which require the preservation of the symmetry of the state. The oscillator strength *f*_*if*_ corresponding to the probability of the one-photon absorption in the electric dipole approximation depends on the energy difference between the involved initial and final states *E*_*f*_ and *E*_*i*_ and the transition dipole moment as follows:

$$f_{if}=\frac{2}{3}\frac{m_{e}}{\hbar^{2}}\left(E_{f}-E_{i}\right)\underset{\alpha =x,y,z}{\sum }|\left\langle \psi_{i}|R_{\alpha }|\psi_{f}\right\rangle {|}^{2},$$ (3)

where *m*_e_ denotes the electron mass, *ℏ* is the reduced Planck constant, and the sum runs over the Cartesian coordinates.

The intersystem crossing rate depends on the spin–orbit coupling matrix elements and is inversely proportional to the singlet–triplet energy gap *E*_T_−*E*_S_ for the involved states. Due to the artificial geometry of the investigated complexes, only the singlet–triplet energy gap is calculated in the present study for the estimation of the influence of the O-by-S replacement and substituent effect in hydrogen-bonded systems on the intersystem crossing.

The two-photon absorption cross section *σ* ^TPA^ is defined in GCS units for the photon energy *ℏω* as follows:

$$\sigma^{\text{TPA}}=\frac{4\pi^{3}\alpha a_{0}^{5}\omega^{2}}{c}\left\langle \delta^{\text{TPA}}\right\rangle g\left(2\omega ,\omega_{0},\Gamma \right),$$ (4)

where *δ* ^TPA^ denotes the TPA strength, calculated for the linearly polarized light as follows:

$$\left\langle \delta^{\text{TPA}}\right\rangle =\frac{1}{15}\underset{ab}{\sum }\left(2S_{ab}{\bar{S}}_{ab}+S_{aa}{\bar{S}}_{bb}\right),$$ (5)

with *S* being the TPA transition moment defined in Beerepoot et al. (2015), and *a* and *b* denoting the Cartesian components. *g* (2*ω*, *ω*_0_, Γ) stands for the lineshape function responsible for inclusion of spectral broadening effects, *α* is the fine structure constant, and *a*_0_, the Bohr radius.

Geometry optimization, supermolecular interaction energy, and one-photon absorption calculations have been performed with the Gaussian16 package (Frisch et al., 2016). Two-photon absorption cross sections have been determined with the Dalton2018 program package (Aidas et al., 2018; Aidas et al., 2014). The non-covalent interaction analysis has been performed within NCIPlot software (Boto et al., 2020; Johnson et al., 2010; Contreras-Garcia et al., 2011) and AIMAll (Keith, 2019; Bader, 1990; Popelier, 2000), and charge transfer indexes have been calculated with Multiwfn3.8 (Lu and Chen, 2021. LPNO-CCSD(T) counterpoise-corrected interaction energies have been obtained within the ORCA 4.2.1 package (Neese et al., 2020a; Neese et al., 2020b; Neese, 2012; Neese, 2018; Altun et al., 2021).

In all the graphics along the article, blue lines and points denote the oxosquaraine systems, while the green ones cover the thiosquaraines. The darkest shade is applied for the isolated (thio)squaraine, the middle shade for the DPU complexes, while the lightest shade for the DPTU complexes.

## 3 Results

### 3.1 Structural Properties

**FIGURE 2:**
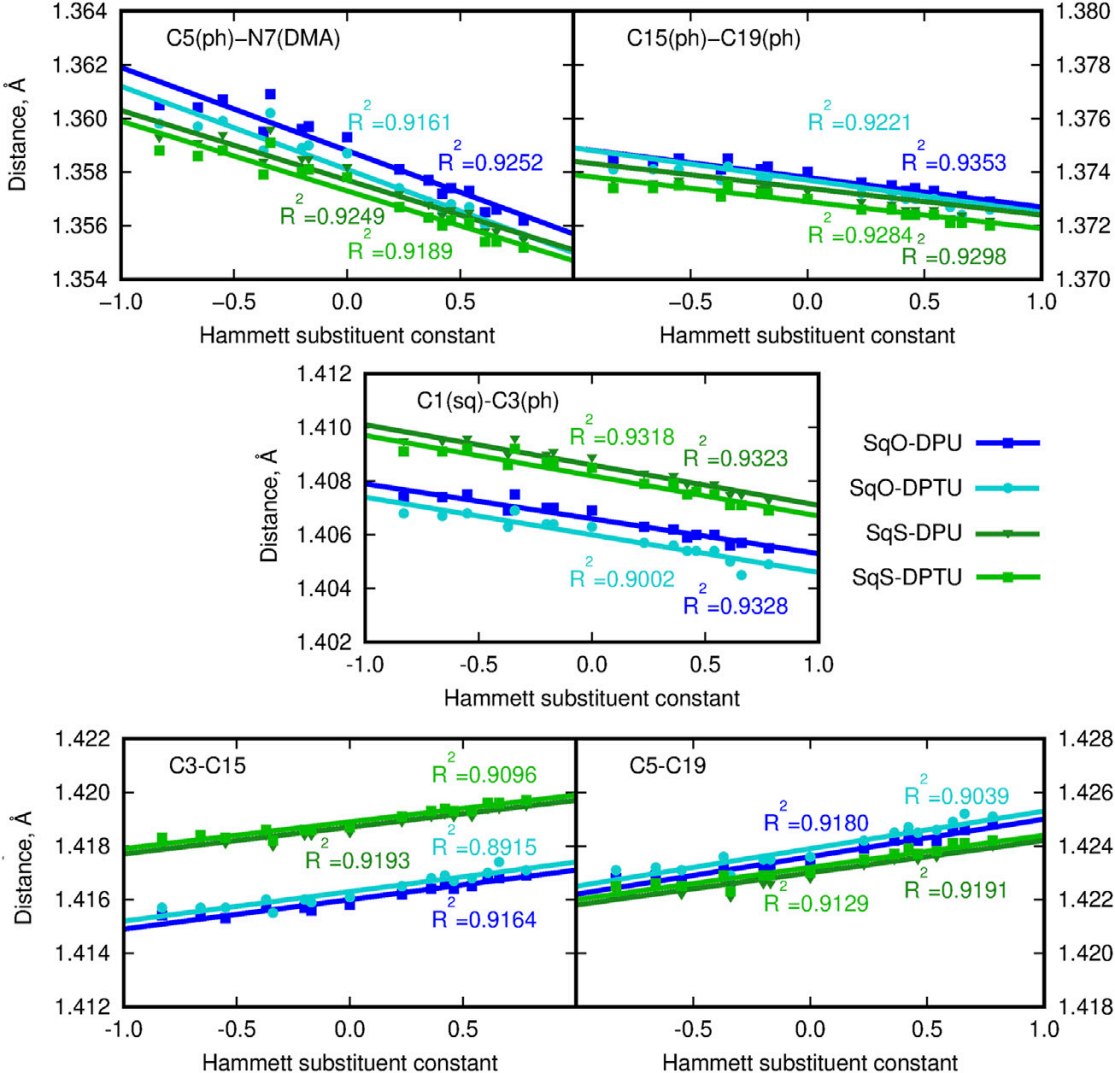
Geometrical parameters for squaraine derivatives (atom numbering in Figure 1B; here and in the article; blue lines/points denote the oxygen-containing squaraine complexes and green lines/points, its thioderivative; while darker lines are used for urea and lighter for thiourea). (DMA) denotes the terminal dimethylamino group and (ph) stands for the phenyl ring in (thio)squaraine.

**FIGURE 3:**
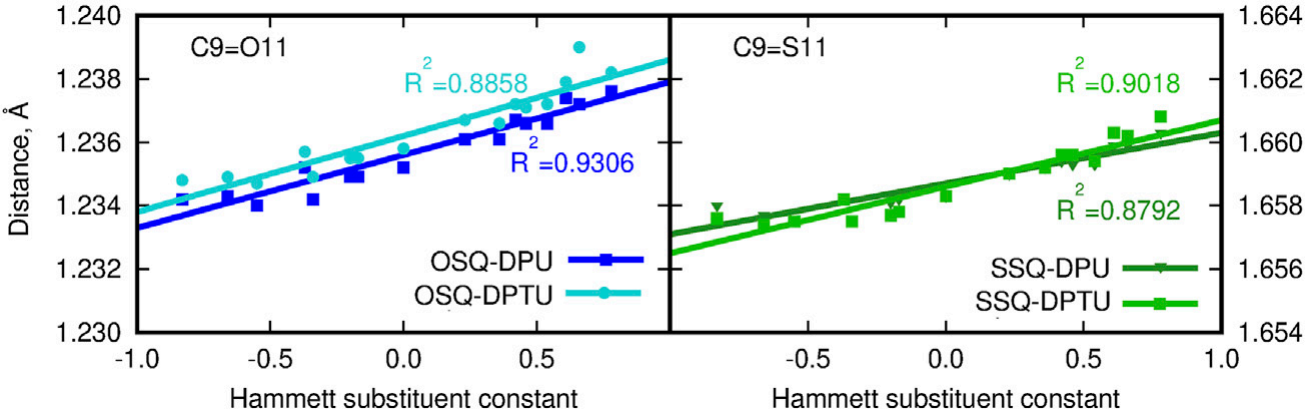
C9-O/S11 bond length for squaraine derivatives (atom numbering in Figure 1B; blue lines/points denote the oxygen-containing squaraine complexes and green lines/points, its thioderivative, while darker lines are used for urea and lighter for thiourea).

Intermolecular interactions of squaraine moiety with the N,N′-diphenyl(thio)urea derivatives affect both the structure of the squaraine scaffold itself and the geometrical parameters of the D–H … A contacts. Figures 2, 3 present the corresponding modification of the bond length in the (thio)squaraine. It can be noticed that the most pronounced modification along the squaraine symmetry axis occurs for the C5–N7 bond (compare Figure 1 for atom numbering), connecting the squaraine phenylene moiety with the -NMe_2_ terminal substituent (Figure 2A). The sequence of the applied urea substituents causes the C5–N7 bond shortening with the growing electron-withdrawing character of the substituent by about 0.004 Å. The corresponding modification in the case of C15–C19 (and equivalent) bonds in the phenylene rings does not exceed 0.002 Å, and for C1–C3 bond, this adjustment remains of the order of 0.003 Å only. These tendencies are independent on the sulfur present either in squaraine itself or in urea. Although small, these differences indicate the stronger bond localization in the systems containing strong EW groups in urea in opposition to the delocalization upon the substitution with strong ED, when the bond length alternation becomes smaller.

**FIGURE 4:**
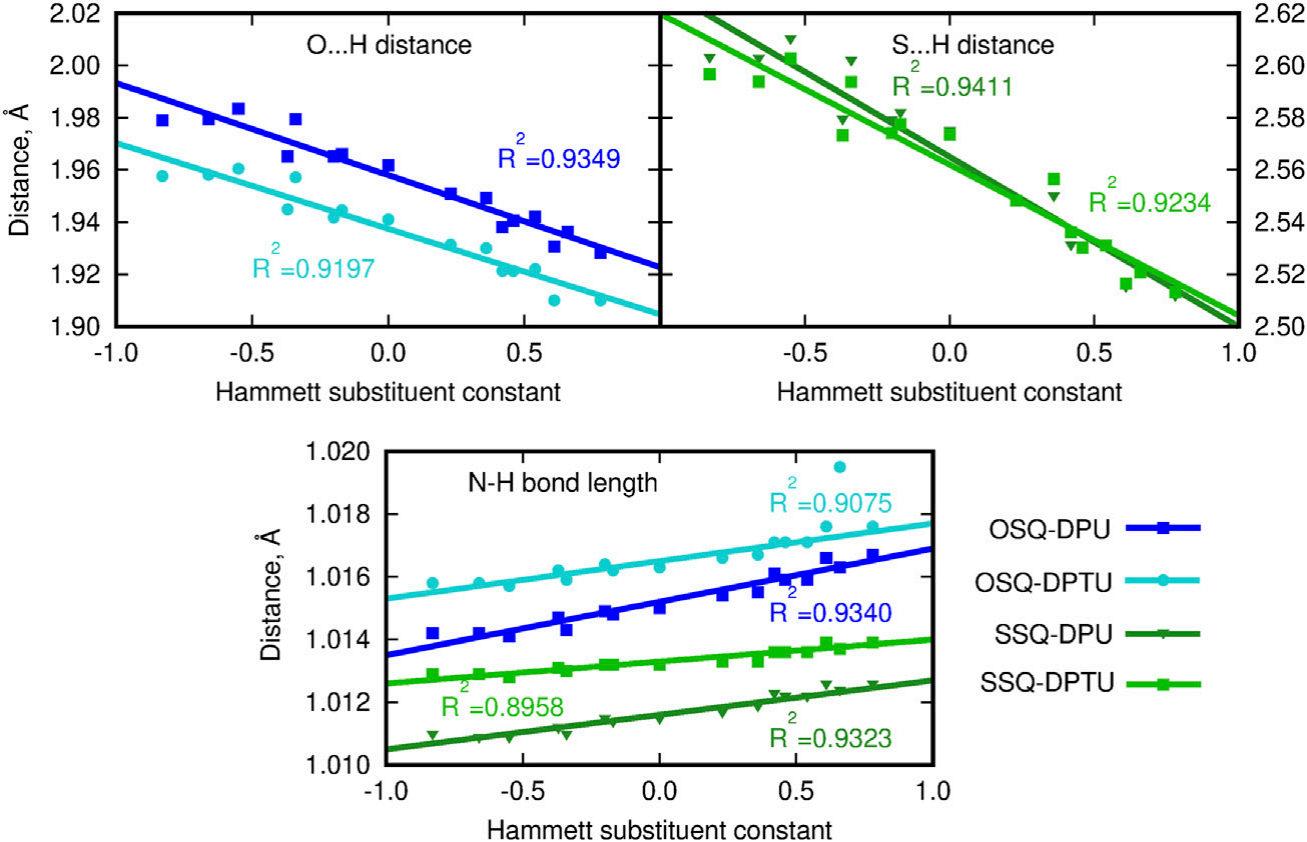
Geometrical parameters for hydrogen bonds (A … H and D–A distances, where A denotes the hydrogen bond acceptor and D, hydrogen bond donor).

On the other hand, the double bond between the C9 carbon in the squaric ring and the O11 oxygen or S11 sulfur atom in OSQ and SSQ, respectively, undergoes modest elongation upon the introduction of the terminal EW group in urea (not exceeding 0.004 Å). Again this elongation is almost not dependent on the sulfur presence either in the urea or in the squaraine. This is the first geometrical indication that the urea substitution with EW groups promotes the O/S … H–N hydrogen bonds shortening and thus strengthening. This observation remains in agreement with the O11/S11 … H (urea) and N (urea)–H (urea) distance modification upon urea substitution, as shown in Figure 4.

Since the size of the sulfur atom is significantly larger than the oxygen one, the scale on both plots is shifted by 0.6 Å for convenience. The O-by-S replacement in squaraine (for urea complexes compare dark blue and dark green lines in upper panels of Figure 4) directly affects the hydrogen bond formed between the subsystems. The sulfur presence in the hydrogen bond makes it more prone to the electronic effects arising from the modification of the substitution pattern, and the hydrogen bond donor … proton distance decreases strongly with the growth of the Hammett substituent constant compared with that in the case of the O … H–N interactions in oxygen-bearing squaraine. This tendency is similar for the influence of the O-by-S replacement in squaraine on the hydrogen bond distance in the thiourea complexes, which can be deduced from the comparable difference of the slopes (see light blue and light green lines in upper panels of Figure 4). The strong electron-withdrawing substituent present in the urea units may cause the shortening of the O … H distance with respect to the most electron-donating group by as much as 0.05 Å in the OSQ complexes and 0.09 Å in SSQ complexes. The effects observed here can be diminished by the considerable size of the squaraine moiety, which prevents the effective substantial intramolecular charge transfer upon the urea substitution. The corresponding modifications for the O … H distance in the formaldehyde–urea and thioformaldehyde–urea complexes are significantly larger, reaching 0.157 9 Å (about 5.5% of the starting value) for the thio derivative of formaldehyde. Therefore, this simple geometrical analysis indicates that the proper design of the supramolecular complexes bearing the chromophore unit allows for the subtle tuning of the electron charge density distribution and thus all of the resulting features.

### 3.2 Charge Distribution in Ground State

**FIGURE 5:**
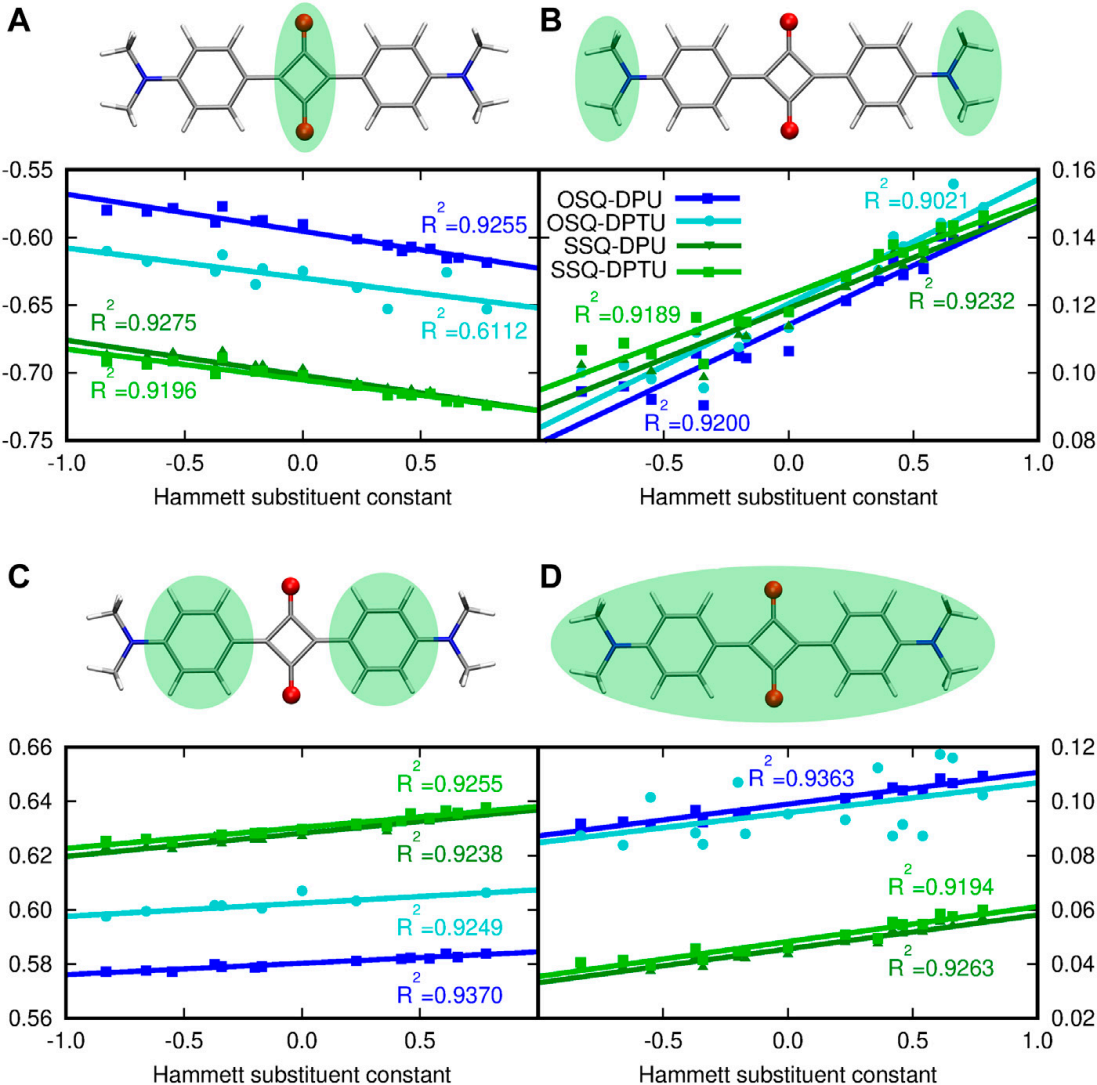
Modification of the NPA partial charge distribution in the central (thio)squaraine unit upon substitution in N,N′-diphenyl(thio)urea fragments: **(A)** in the central squaric ring, **(B)** in the terminal N,N-dimethylamino groups, **(C)** in phenyl rings, and **(D)** in the whole (thio)squaraine moiety. Sum of partial charges from the considered fragment, presented in the green background, is given as an ordinate.

The presence of the ED or EW substituents in the urea derivatives is expected to modify the charge distribution in the urea molecules itself. However it could be also assumed that such an adjustment of the electron density in the hydrogen bond–donating species is strong enough to affect the hydrogen bond, and thus hydrogen bond acceptor and its properties as well. Therefore the charge distribution in the squaraine moiety is presented with respect to the Hammett constant in the substituted N,N′-diphenyl(thio)urea (Figure 5). The sum of NBO charges for the (thio)squaraine, presented in panel (D) of Figure 5, indicates that the interaction in the considered hydrogen-bonded complex can be perceived as a way of drawing the electrons from the squaraine unit. This effect is almost twice as strong in the oxygen-bearing squaraines as for their thio-counterparts. Additionally, the stronger the electron-accepting character of the substituents in N,N′-diphenyl(thio)urea, the more positive the partial charge in squaraine that remains in agreement with chemical intuition. The O-by-S exchange in urea on the other hand affects partial charge in (thio)squaraine only marginally.

The central squaraine system is divided into three parts [panels (A–C) of Figure 5, respectively], according to the character of these parts, namely, the terminal N,N–dimethylamino group serves as the strong electron donor and the squaric ring in the center is an electron-withdrawing moiety, while phenyl rings provide the scaffold enabling communication between those moieties. Introduction of the electron-donating substituents in the N,N′-diphenyl(thio)urea causes the decrease in the negative charge cumulated in the central squaric ring, while the electron-accepting groups present in the N,N′-diphenyl(thio)urea leads to the increase of the negative charge in this part of the system. The opposite trend can be noticed for the terminal N,N-dimethylamino group, which gather the negative charge in the case of the ED group introduced in the N,N′-diphenyl(thio)urea and give it back to the squaric ring in the case of EW substituent. Moreover, at the N,N-dimethylamino groups the charge redistribution in squaraine moiety seems to be most spectacular, since the charge increases by about 50% with the growth of the Hammett constant, depending on the system [compare panel (B) in Figure 5]. The O-by-S substitution both in squaraine and in urea units does not affect the general tendencies for the charge distribution particularly for the terminal N,N-dimethylamino groups. In the case of the central squaric ring, the sulfur presence in the thiosquaraine strongly increases the negative charge gathered by the squaric ring in comparison to the pristine oxosquaraine, while the O-by-S exchange in urea is noticed also to increase the negative charge accumulated in the central part of the complex, but only tiny effect is observed. On the other hand, for the thiosquaraine, the presence of the sulfur in the thiourea moiety does not introduce any further meaningful modifications. Additionally, the Le Bahers charge transfer indexes show that due to the centosymmetric architecture of the investigated systems, the general shift of the charge distribution upon excitation is equal to zero. Moreover, the Δ*σ* parameter indicates that the effect is tiny and smaller for the thiosquaraine complexes than for the oxosquaraine ones.

### 3.3 Substituent Effects in the Interaction Energy

**FIGURE 6:**
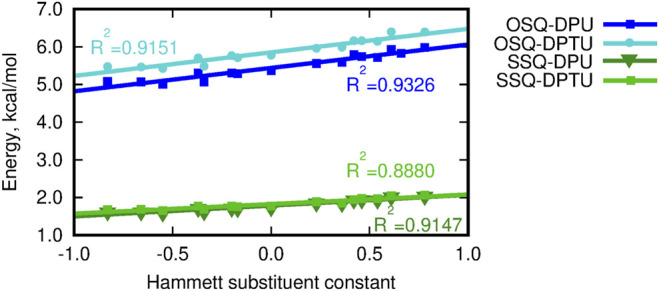
Hydrogen bond energy estimated according to Espinosa et al. (1998) versus the Hammett substituent constant.

**FIGURE 7:**
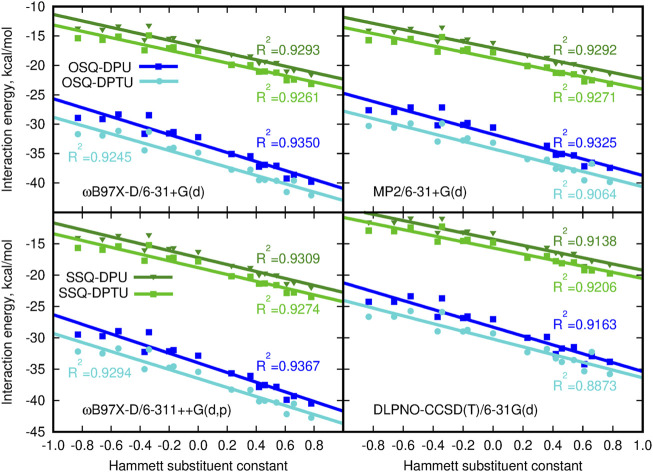
Counterpoise-corrected supermolecular interaction energy for the three-component system calculated according to the site–site counterpoise scheme versus the Hammett substituent constant.

The dependence of the supermolecular interaction energy on the Hammett constant of the substituent in urea derivative, estimated within the DFT, MP2, and DLPNO-CCSD(T) approaches, is presented in Figure 7. The blue lines denote the OSQ complexes, while the green ones, the SSQ systems. One can see that the general tendencies are independent on the methodology applied and hold for all of the employed methods. First of all, the interaction is twice as large for the pristine, oxygen-containing squaraines as for their thio-analogs, and this difference is larger than 15 kcal/mol for all analyzed cases. This can be expected due to the known larger strength of the N–H … O hydrogen bonds than the N–H … S ones. This trend is confirmed by the hydrogen bond strength, *E*_HB_, estimated according to Espinosa et al. (1998) as the negative of the half of the potential energy density (virial field) within AIM (Bader, 1990; Popelier, 2000) (see Figure 6 and Supplementary Material). *E*_HB_ is roughly three times larger for the oxygen-containing squaraines than for the thiosquaraines (lower panel in Figure 6). On the other hand, the O-by-S replacement in the diphenylurea species leads only to the tiny enhancement of the attraction. The introduction of sulfur atoms to the urea derivatives amplifies the H-bonds up to 10% that arises from the larger acidity of the N–H protons in thiourea than in urea. This makes the difference of the order of 0.5 kcal/mol for oxosquaraine complexes and less than 0.1 kcal/mol for thiosquaraine ones. All of the lines plotted in panels of Figure 7 remain almost parallel, which means that the substituent effect is the same in both series. However, the stronger the electron-accepting character of the substituent is, the interaction energy becomes more attractive, since the urea withdraws the electrons from the central squaraine moiety, thus making the hydrogen bond stronger. These effects are slightly more pronounced for oxygen-bearing squaraines (attraction enhancement by about 10 kcal/mol) than for their thio-analogs (7–8 kcal/mol).

**FIGURE 8:**
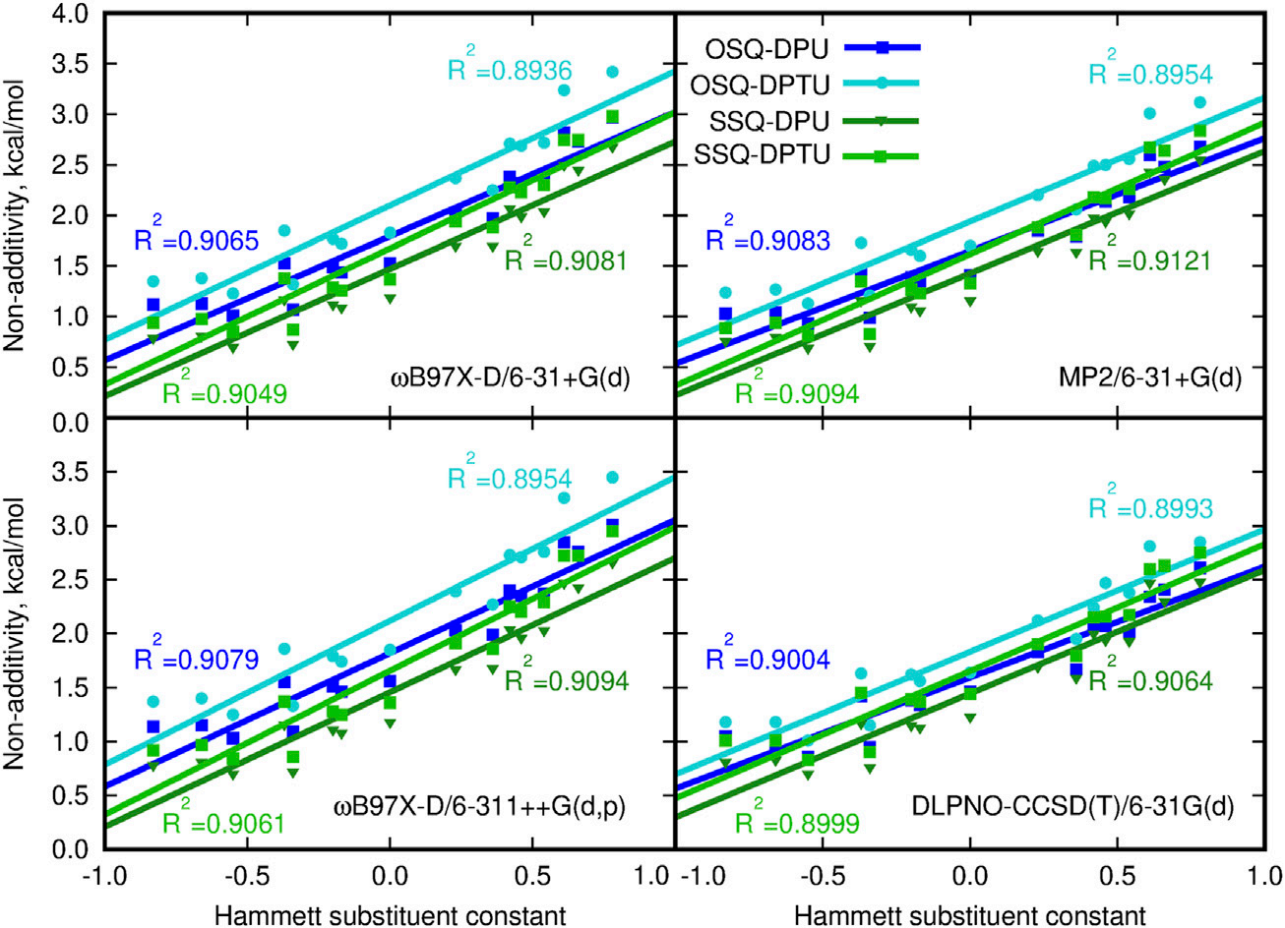
Non-additivity in the supermolecular interaction energy versus the Hammett substituent constant.

It is interesting to investigate to what extent the interactions in OSQ and SSQ complexes with DPU originate from extra interaction, beyond pairwise molecule–molecule interactions. In other words, it is of importance if there is any cooperative effect in the complex which cannot be reproduced merely by adding SQ molecules and DPU units. The significance of the three-body effect for the total interaction is presented in Figure 8. The observed effects are destabilizing: inclusion of the third subsystem in the complex decreases the mutual attraction observed when only two-body terms are taken into account. The non-additivity is of similar order of magnitude for all of the investigated series of systems and clearly exhibits the dependence on the substituent character. These effects equal to about 1 kcal/mol for the most electron-donating N,N-dimethylamino group and increasing to about 2.5–3.5 kcal/mol for the most electron-withdrawing nitro group and are slightly higher for the oxosquaraine than for its thio-counterpart. It should be also noticed that beside the enhancement of the absolute value of the three-body effects, its percentage into the total supermolecular interaction energy is doubled within the analyzed substituent series for all of the oxo- and thio-complexes. However here, due to the significantly weaker interaction in thiosquaraine systems than in oxosquaraine ones, the overall percentage of the three-body effects is larger for squaraine-containing sulfur atoms and reaches from 5.5% for -NMe_2_ substituent to 12.5% for -NO_2_. Notwithstanding, the magnitude of three-body effects in these complexes is relatively small, which allows for simplified treatment of bigger analogs of these molecules.

**FIGURE 9:**
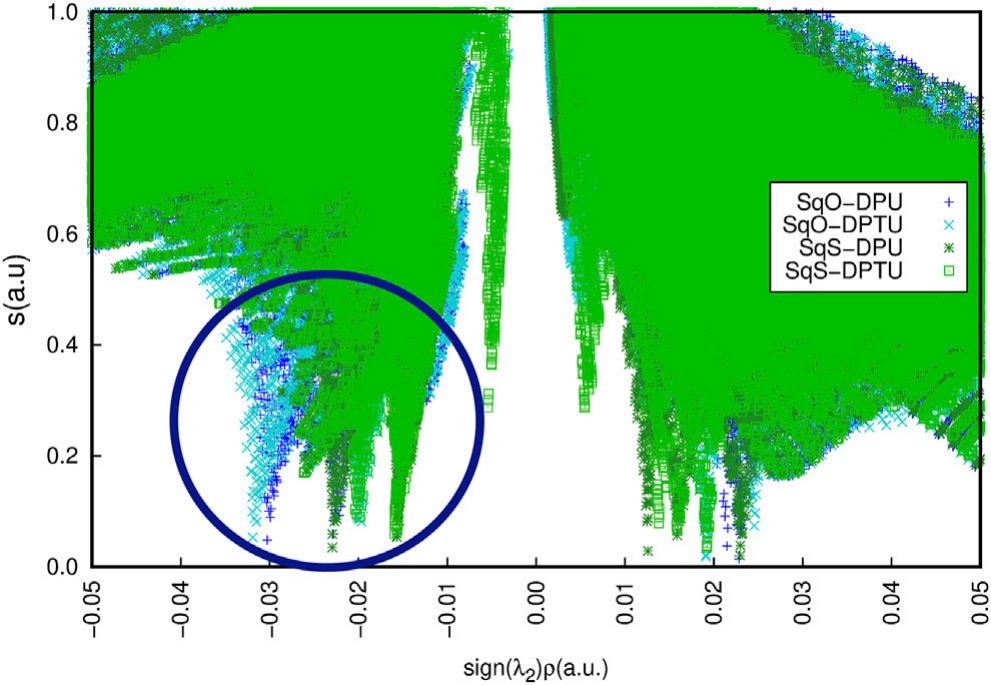
Non-covalent interactions for the unsubstituted systems according to the NCIPlot analysis for OSQ-DPU (blue), OSQ-DPTU (turquoise), SSQ-DPU (dark green), and SSQ-DPTU (light green).

Additionally, the tiny difference for OSQ and SSQ complexes in the character of the interactions can be noticed by the NCI analysis (Figure 9). The difference observed in the range of −0.035 to −0.02 a. u. (marked by the navy oval) concerns the character of the contacts responsible for the hydrogen bonds.

### 3.4 Substituent Effect on the Orbital Energy Levels

Since the introduction of the sulfur into the system is known to insert additional energy levels arising from the lone pairs of sulfur, it is expected to provide a desired tool for tailoring of the chromophores properties. Thus, the sulfur presence in the squaric ring leads to the inversion of the lowest *π* → *π*∗ and *n* → *π*∗ excitations in this manner decreasing the singlet–triplet energy gap (Webster et al., 2010; Avirah et al., 2012; Peceli et al., 2013). This can be a way of increasing the intersystem crossing for the efficient application of squaraine triplet states, for instance, in photodynamic therapy or in phosphorescent molecular devices.

**FIGURE 10:**
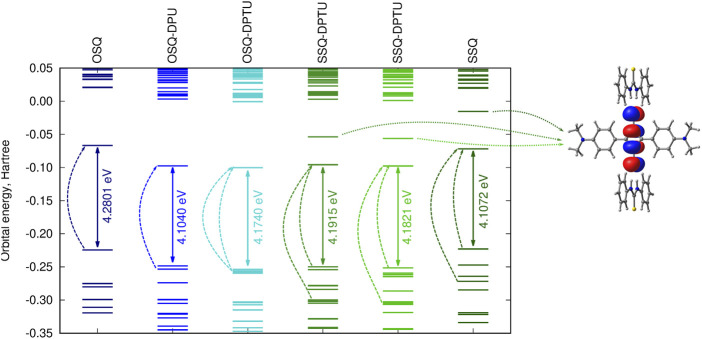
Orbital energy levels [CAM-B3LYP/6-31+G(d)] for the investigated unsubstituted systems together with the LUMO+1 orbital for thiosquaraine (dashed lines indicate the orbitals involved in the most intensive one-photon singlet–singlet transitions; for the corresponding orbital shapes compare Figure 12).

**FIGURE 11:**
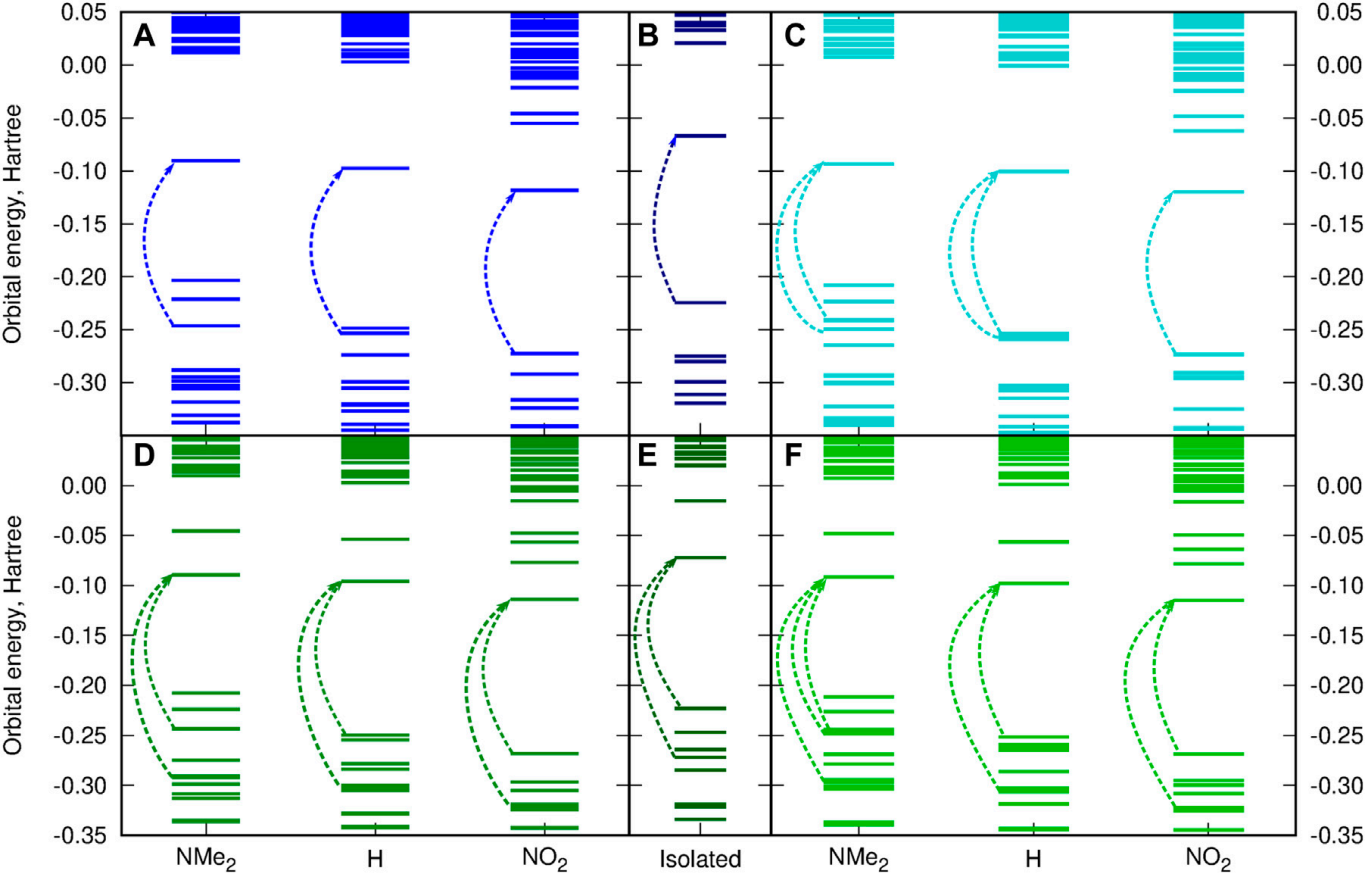
Substitution effect on the orbital energy levels [CAM-B3LYP/6-31+G(d)] for the investigated systems in comparison to the isolated (thio)squaraine **(A)** OSQ-DPU, **(B)** isolated OSQ, **(C)** OSQ-DPTU, **(D)** SSQ-DPU, **(E)** isolated SSQ, and **(F)** SSQ-DPTU (dashed arrows indicate orbitals involved in most intensive long-wavelength transitions).

**FIGURE 12:**
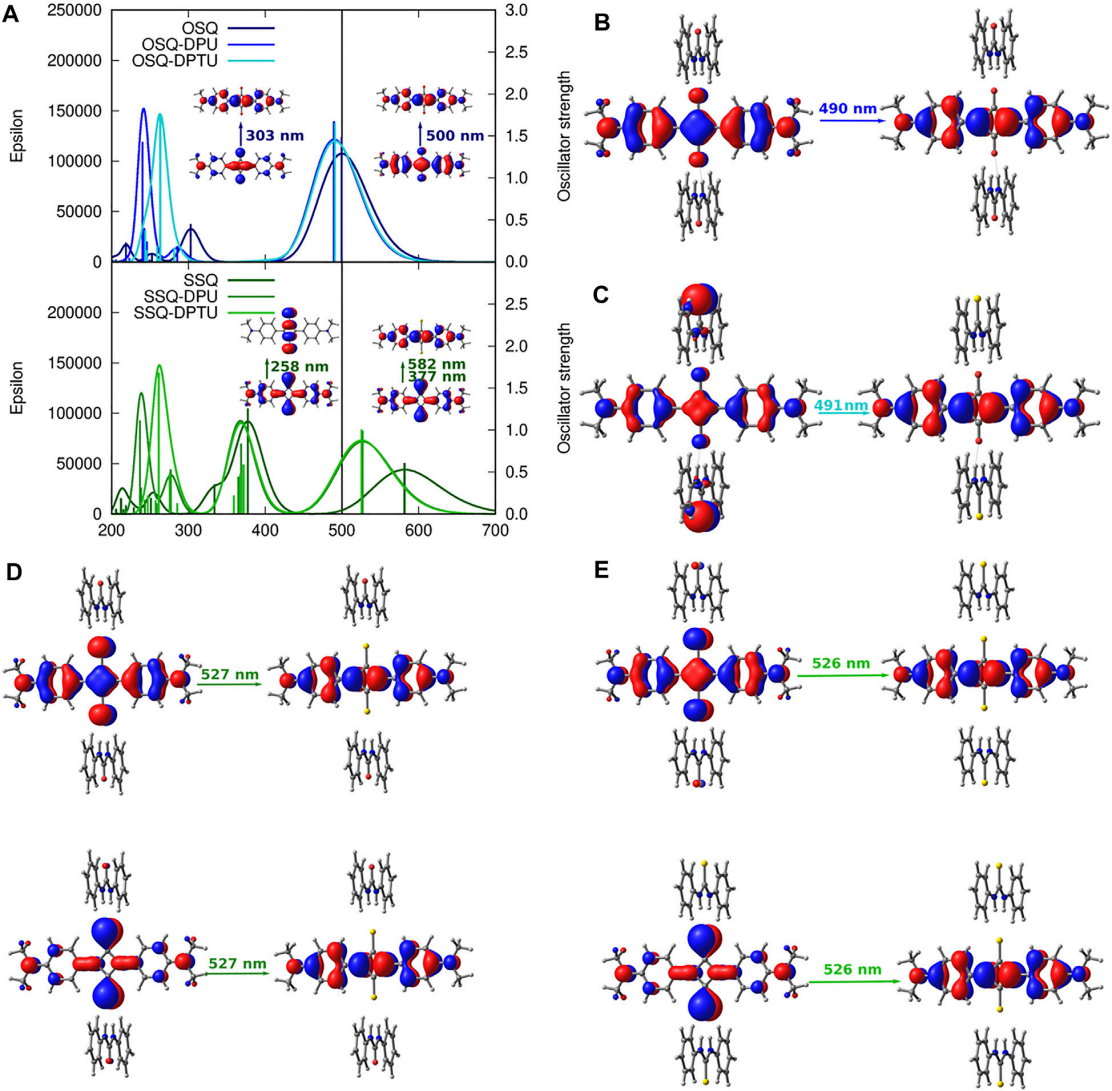
One-photon absorption [CAM-B3LYP/6-31+G(d)] together with the corresponding orbitals for investigated systems: **(A)** vertical absorption spectrum for oxosquaraine and its complexes **(upper panel)** and thiosqaraine **(lower panel)**, **(B)** OSQ-DPU frontier orbitals, **(C)** OSQ-DPTU frontier orbitals, **(D)** SSQ-DPU frontier orbitals, and **(E)** SSQ-DPTU frontier orbitals (convolution of the theoretical oscillator strengths with Gaussian function for full-with half-maximum equal to 3,000 cm^−1^).

Figure 10 presents the frontier orbital energy levels for the isolated squaraine and thiosquaraine in comparison to their N,N′-diphenyl(thio)urea complexes. One needs to carefully pay attention to the orbitals involved in the most intensive long-range singlet–singlet transition—not for all of these systems, these are simply HOMO and LUMO orbitals, as pointed out by dashed lines in Figure 10. However, in all of the investigated systems, the type of the orbitals involved in excitations is preserved: the transition occurs from and to the orbitals localized mainly or even exclusively in the (thio)squaraine moiety (compare Figure 12). In the case of the thiosquaraines, additionally the lone pairs on the sulfur are involved. Although their contribution to the excitation is minor, the energy difference between this lone pair and LUMO undergo the uplifting upon the (thio)urea substitution even by one order of magnitude more that for the corresponding *π* → *π*∗ transitions (0.003 49 vs. 0.000 32 Hartree for SSQ-DPU complexes, compare Figure 11). The highest occupied molecular orbitals for the -NMe_2_ substituted systems correspond to the N,N′-diphenyl(thio)urea *π* orbitals involved in the transitions in the UV range below 300 nm (see Figure 12). Taking the orbital energy difference as the first approach to the electronic excitation spectrum, one can clearly foresee the differences between the oxosquaraine and thiosquaraine complexes absorption upon introduction of EW/ED groups in the N,N′-diphenyl(thio)urea.

### 3.5 Substituent Effect on the One-Photon Absorption

One-photon absorption for the investigated complexes is expected to undergo modifications arising from the introduction of the heavy (sulfur) atom and/or EW/ED substituents due to the changes in the orbital picture presented in the previous section. The electronic absorption spectrum of thiosquaraine differs from the oxygenated squaraine by the broader and red-shifted lower intensity absorption band in the long wavelength range (582 nm for isolated thiosquaraine vs. 500 nm for oxosquaraine, as presented in Figure 12), and the signal at 303 nm observed for isolated oxosquaraine is shifted to 377 nm for isolated thiosquaraine (compare Supplementary Material).

**FIGURE 13:**
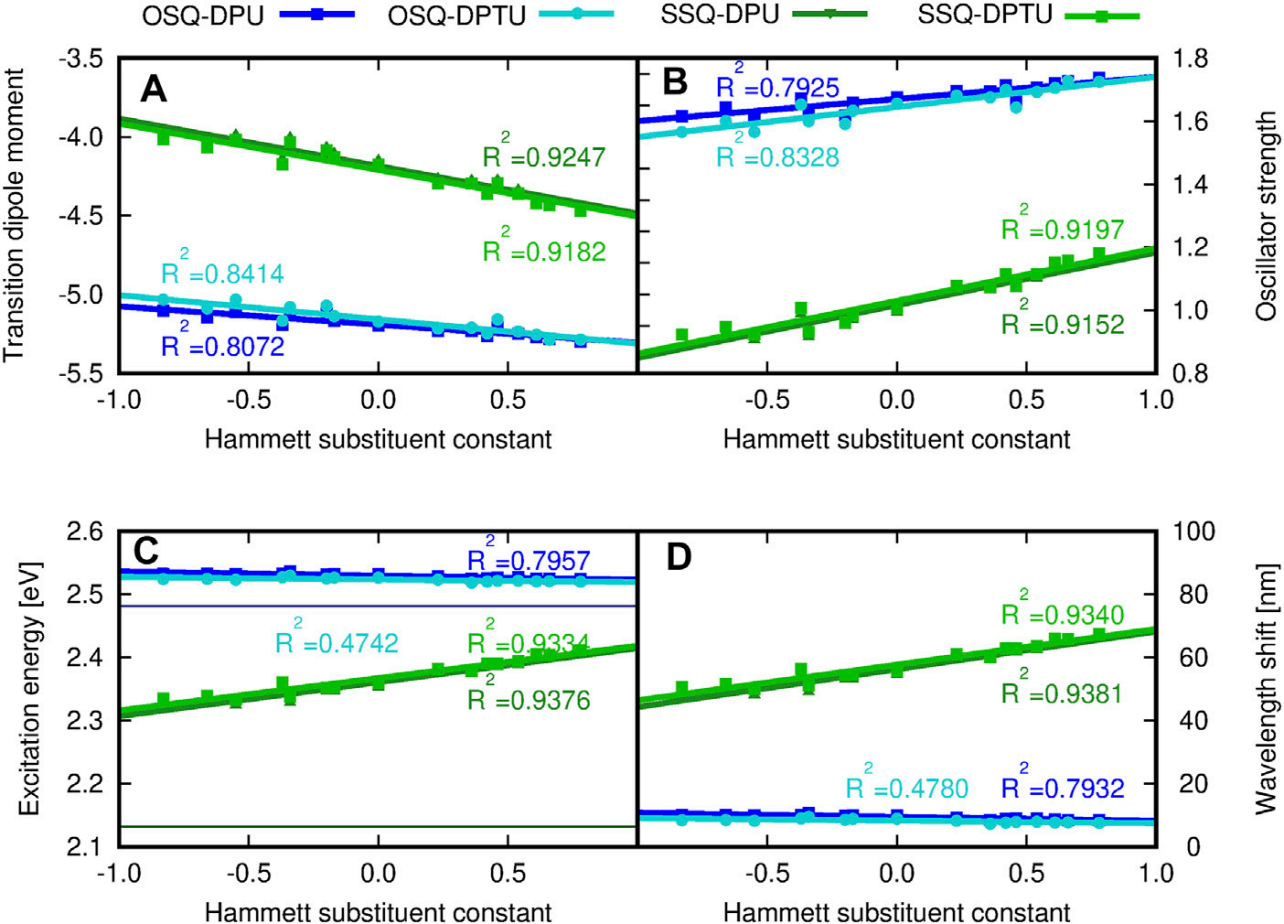
**(A)** Transition dipole moment component, **(B)** oscillator strengths, **(C)** excitation energy (eV), and **(D)** maximum wavelength shift (nm) with respect to the isolated oxo- or thiosquaraine spectrum, respectively, for one-photon absorption in the investigated complexes versus the Hammett substituent constant for substituents in N,N′-diphenyl(thio)urea estimated within the CAM-B3LYP/6-31+G(d) approach. The reference excitation energy for isolated squaraines is shown for comparison in panel **(C)** as a thin navy horizontal line for oxosquaraine and thin dark green horizontal line for thiosquaraine.

Figure 13 depicts the general tendencies in the OPA spectrum for investigated complexes upon substitution [panels (A–C)] and the comparison of the (thio)squaraine-in-complex with the isolated (thio)squaraine [panel (D)]. The maximum absorption wavelength in complex is shifted with respect to the isolated species by only tiny margin by 7–11 nm for oxosquaraines and significantly by almost 50–70 nm for thiosquaraines [Figure 13, panel (D)]. Clearly, the spectrum modification with respect to the isolated (thio)squaraine upon N,N′-diphenyl(thio)urea substitution is also almost constant for oxosquaraines. On the other hand, for thiosquaraines, the maximum absorption wavelength shift depends strongly on the introduction of the substituents in the urea. Thus, the total effect of the spectrum adjustment upon interactions can be roughly divided into two components: one arising from the complex formation by hydrogen bonds to unsubstituted urea derivatives and the other from the substitution of the urea phenyl rings. The influence of the complex formation *via* hydrogen bonds with the unsubstituted N,N′-diphenyl(thio)urea causes the bathochromic shift of absorption spectrum by 9–10 nm for oxosquaraine and by 54.5–56 nm for thiosquaraine. Additionally, upon hydrogen bonding, a gentle increase of the band intensity is noticed. Further substitution of urea units leads to the additional shifts on less than 2 nm for oxosquaraines and up to 12 nm for thiosquaraines. Therefore, the O-by-S replacement in squaraine is expected to provide a way of achieving the strong bathochromic shift of absorption spectrum, the formation of hydrogen-bonded complexes particularly in the case of thiosquaraine produces also the significant red-shift, and the tiny tailoring of the absorption spectra can be reached by the proper substitution on the phenyl rings of urea. Moreover, the thionylation of the diphenylurea is negligible both for oxosquaraine and thiosquaraine absorption spectrum.

The stronger shift in the thiosquaraine than for oxosquaraine can be ascribed to the lone-pair sulfur orbitals more prone to the influence made by the EW/ED substituents than the regular *π* orbitals involved in the excitations in the case of oxosquaraine.

Moreover, it should be noticed that the O-by-S replacement in squaric ring also affects the oscillator strength *f*, determining the probability of the transition [compare panel (B) of Figure 13]. The complexes of thiosquaraine exhibit significantly higher values of *f*, additionally increasing with the growing substituent Hammett constant for the groups present in N,N′-diphenyl(thio)urea units.

**FIGURE 14:**
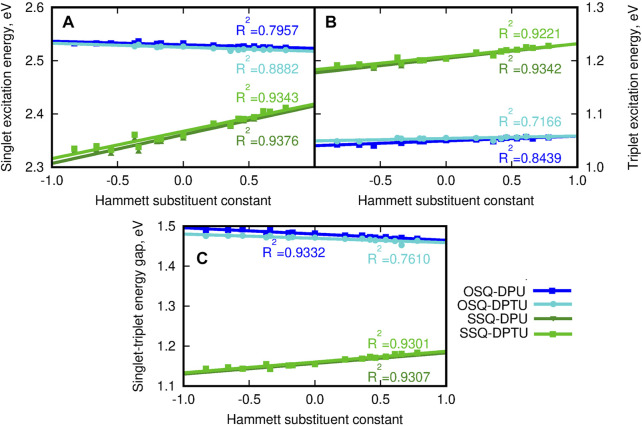
**(A)** Vertical singlet excitation energy (eV), **(B)** vertical triplet excitation energy (eV), **(C)** singlet–triplet energy gap (eV) for the investigated complexes versus the Hammett substituent constant for substituents in N,N′-diphenyl(thio)urea estimated within the CAM-B3LYP/6-31+G(d) approach. The reference triplet excitation energies for isolated squaraines are equal 0.805 4 eV and 1.028 0 eV, respectively, for oxo- and thiosquaraine; for the reference singlet excitation energy see panel **(C)** of Figure 13.

Since the sulfur presence in the squaric ring is known to invert the ordering of the states and reduce the singlet–triplet energy gap, the vertical triplet state energy and corresponding singlet–triplet energy gaps have been determined for the considered systems and depicted in Figure 14 (compare also Supplementary Material). The singlet excited state for the thiosquaraine complexes is characterized by the lower energy than for the oxosquaraine ones, and the tendency observed for the triplet states is opposite: the O-by-S replacement in the squaric ring causes the elevation of the excitation energy. This remains in agreement with the expectations based on the literature reports (Webster et al., 2010; Avirah et al., 2012; Peceli et al., 2013; Pristash et al., 2020; Chetti et al., 2021). One should also notice that the excitation energy for the oxosquaraine systems both of singlet and triplet states persists almost constant independently on the substitution pattern in N,N′-diphenyl(thio)urea units, while in the case of the thiosquaraine complexes, the growing electron-withdrawing character of the substituent leads to the stronger increase of the excitation energy for singlet and triplet states. Thus, the urea substitution can be perceived also as the way of fine modification of the singlet–triplet energy gap for hydrogen-bonded thiosquaraines and—in this way—the tool to manipulate the probability of the intersystem crossing.

### 3.6 Two-Photon Absorption

**FIGURE 15:**
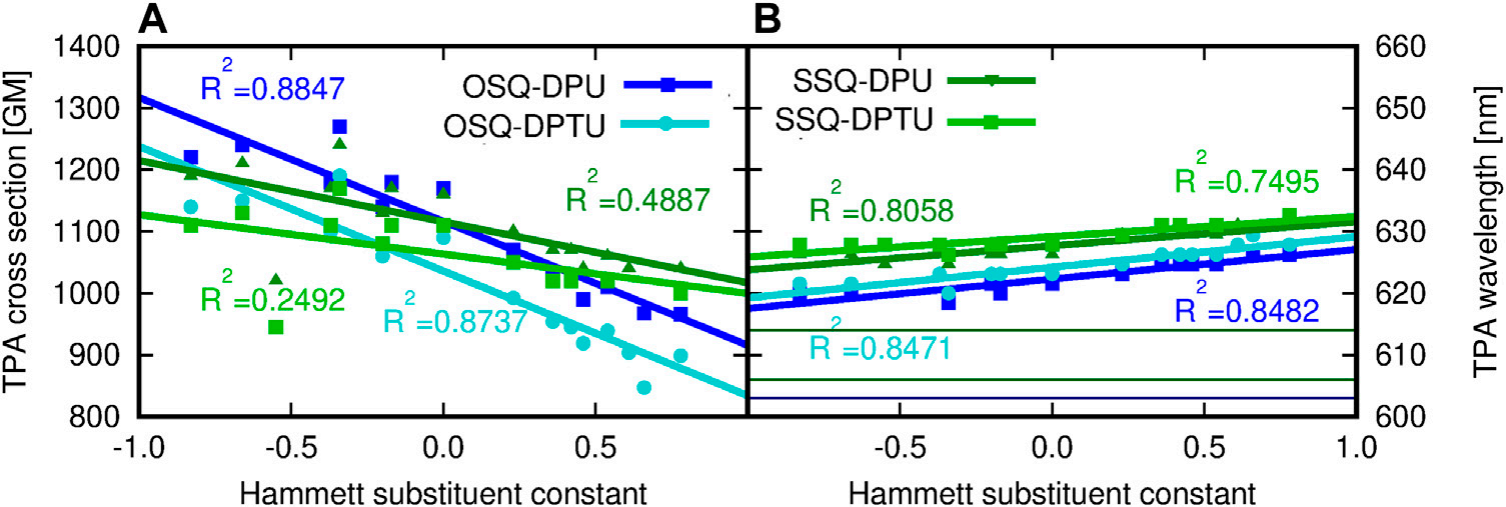
**(A)** Two-photon absorption cross section (GM) and **(B)** two-photon absorption wavelength (nm) for the investigated complexes versus the Hammett substituent constant, determined within the CAM-B3LYP/6-31+G(d) approach (for comparison, the TPA wavelength of isolated species are equal to 603 and 614 nm for OSQ and SSQ, respectively).

Comparison of the reference values for the isolated oxo- and thiosqaraine indicates the bathochromic shift of the TPA wavelength by 11 nm upon O-by-S replacement in the squaric ring accompanied by the slight decrease of the high TPA cross section which remains in agreement with literature reports considering similar oxo- and thiosquaraine derivatives Webster et al. (2010); Avirah et al. (2012). However, for both isolated systems, the *σ* ^TPA^ values are still tremendous and this makes them interesting agents for TPA applications. Therefore, the possibility of the fine-tuning of the TPA wavelength together with controlling the intensity of the signals remains vital. Hydrogen bonding of squaraine derivatives with N,N′-diphenylurea influences its TPA, as it is illustrated in Figure 15. The hydrogen bond leads to the shift of 2*λ* to 622 nm for oxosquaraine (by 19 nm) and 614–626 nm (by 12 nm) for thiosquaraine, thus shifting these values more into the therapeutic window. Such supramolecular architecture involving the hydrogen bonds additionally strongly diminishes the signal intensity (namely, TPA cross section from 2010 to 1090 GM for oxosquaraine and from 1540 to 1160 GM for thiosquaraine); however, these values are still large and promising for applications. The TPA wavelength (2*λ*) is red-shifted with the increasing electron-withdrawing character of the diphenylurea substituent, and the shift is mild both for oxosquaraine (6 nm: from 620 nm for NMe_2_ derivative by 622 nm for unsubstituted diphenylurea to 626 nm for NO_2_ derivative) and for thiosquaraine (5 nm: 626 nm, 626 and 631 nm, respectively). Additionally, the intensity of the TPA increases with the growing electron-donating character of the substituent from 966 GM for nitro-diphenylurea to 1220 GM for dimethyloamino-diphenylurea derivative hydrogen bonded to oxosquaraine. The striking difference in the substituent influence on the OPA and TPA of oxosquaraine can be noticed in comparison to the thiosquaraine case. For oxosquaraine, modification of the EW/ED character of N,N′-diphenyl(thio)urea substituent in fact does not affect the singlet excitation energy (*λ* shift smaller than 2 nm), while the TPA wavelength (2*λ*) is shifted more than 6 nm. These effects remain tiny, however, opposite to those observed thiosquaraine complexes, where OPA wavelength can be modified as much as by 17 nm, and the effect of substitution on TPA wavelength becomes smaller than for oxosquaraine systems (below 5 nm).

From Figure 15, it may be concluded that the oxygen in squaraine is preferred when one need to tune the TPA cross-sectional value by hydrogen bonding. That is caused not only by higher electronegativity of the oxygen atom and higher CT character toward the center of OSQ than that in SSQ but also by the stronger H-bonding of oxygen as an acceptor of hydrogen bond. On the other hand the tuning of the position of the absorption can be realized by both compounds with preference for sulfur if one aims at the red-shifted spectra. Final remark and the challenge for synthesis are to obtain the monooxo derivatives containing one O and one S atom.

## 4 Discussion

The present study is devoted to the development of the controlled modifications of photophysical properties of model squaraine dye. The investigated features such as one-photon absorption, two-photon absorption, or singlet–triplet energy gap affecting the intersystem crossing efficiency are shown to be prone to the fine-tuning by O-by-S replacement in the squaric ring of the chromophore by symmetric hydrogen bonding, by N,N′-diphenyl(thio)urea, and even by systematic modification of the character of substituents present in this N,N′-diphenyl-(thio)urea units. Meaningful differences are observed yet for isolated oxo- and thiosquaraines, namely, the strong bathochromic shift of one-photon absorption or the singlet–triplet energy gap arising from the reversed order of the orbitals involved due to the presence of sulfur, which has been reported earlier. However, this O-by-S replacement is shown to also affect the susceptibility of the central squaraine moiety to supramolecular adjustments arising from hydrogen bonding. Thiosquaraines exhibit significant one-photon absorption blueshift upon hydrogen bonding with the growing electron-withdrawing character of the substituent present in position 4 of N,N′-diphenyl(thio)urea units, in opposition to the weak effects observed for oxosquaraines. On the other hand, the substituent effect in the hydrogen bond donor is similar in case of two-photon absorption for both oxo- and thiosquaraines. A strong reduction of singlet–triplet gap upon O-by-S replacement in squaraines can be further adjusted by hydrogen bonding and introduction of electron-donating substituents in urea. Thus, the present study provides a controlled way of modifying photophysical properties of squaraine dyes by the series of factors (O-by-S replacement, hydrogen bonding, substituent effects in hydrogen bond donor) and indicates the strong and gentle outcomes which allow to precisely adjust the desired features of system of interest, when properly combined.

## Data Availability

The original contributions presented in the study are included in the article/Supplementary Material; further inquiries can be directed to the corresponding author.
