# Supplementary material for: Supramolecular Approach to Tuning the Photophysical Properties of Quadrupolar Squaraines
Source: Front Chem. 2022 Jan 5;9:800541. doi: 10.3389/fchem.2021.800541 (PMC8766669; doi:10.3389/fchem.2021.800541)
Supplement: Supplementary file 1 [file DataSheet1.pdf]

Supporting Information: Supramolecular approach to tuning the photophysical properties of quadrupolar squaraines

Anna Kaczmarek-Kędziera,*,† Borys Ośmiałowski,† Piotr S. Żuchowski,‡ and Dariusz Kędziera†

†*Faculty of Chemistry, Nicolaus Copernicus University in Torun, Gagarina 7, 87–100 Toruń, Poland*

‡*Institute of Physics, Nicolaus Copernicus University in Torun, Grudziądzka 5, 87–100 Toruń, Poland*

E-mail: teoadk@chem.umk.pl

December 14, 2021

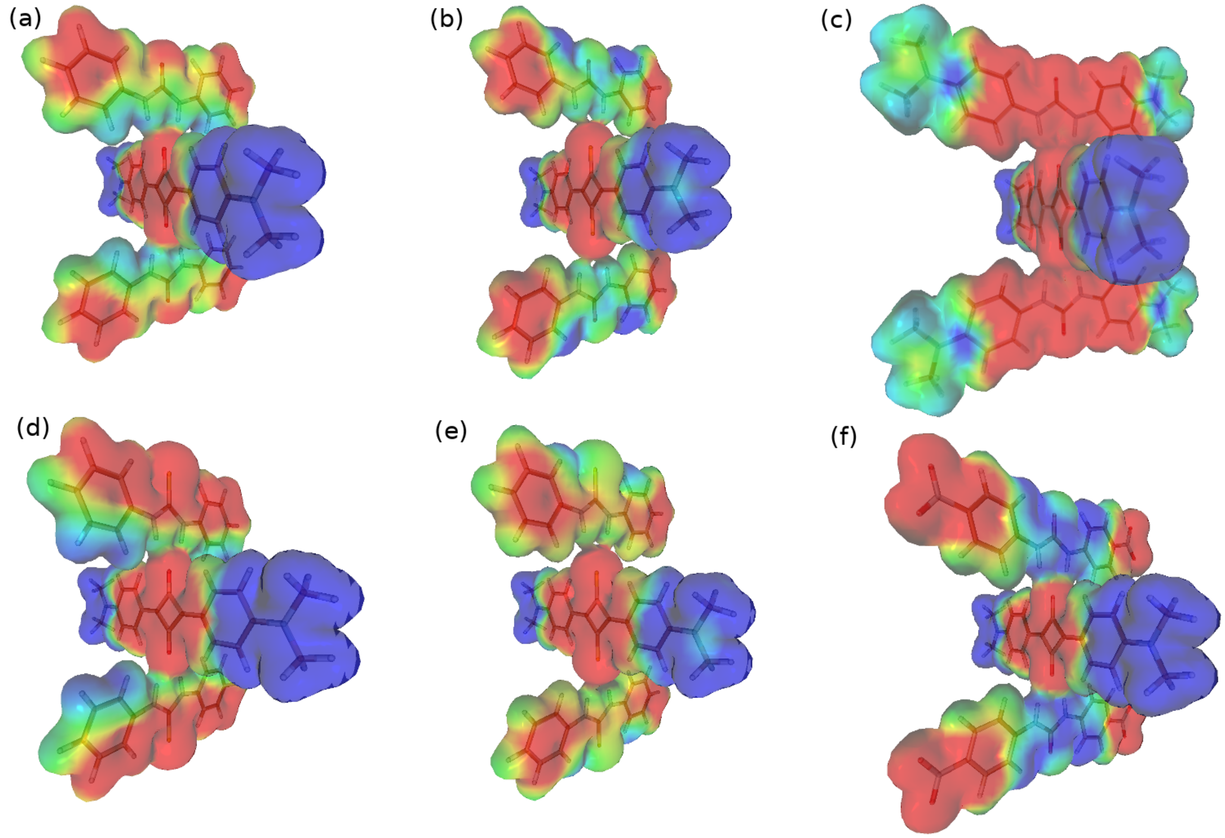

Figure 1: Electrostatic potential of hydrogen-bonded squaraine and thiosquaraine complexes with two N,N'-diphenylurea molecules and N,N'-diphenylthiourea molecules and their substituted analogs: (a) OSQ-DPU, (b) SSQ-DPU, (c) OSQ-DPU(NMe_2), (d) OSQ-DPTU, (e) SSQ-DPTU, (f) OSQ-DPU(NO_2)

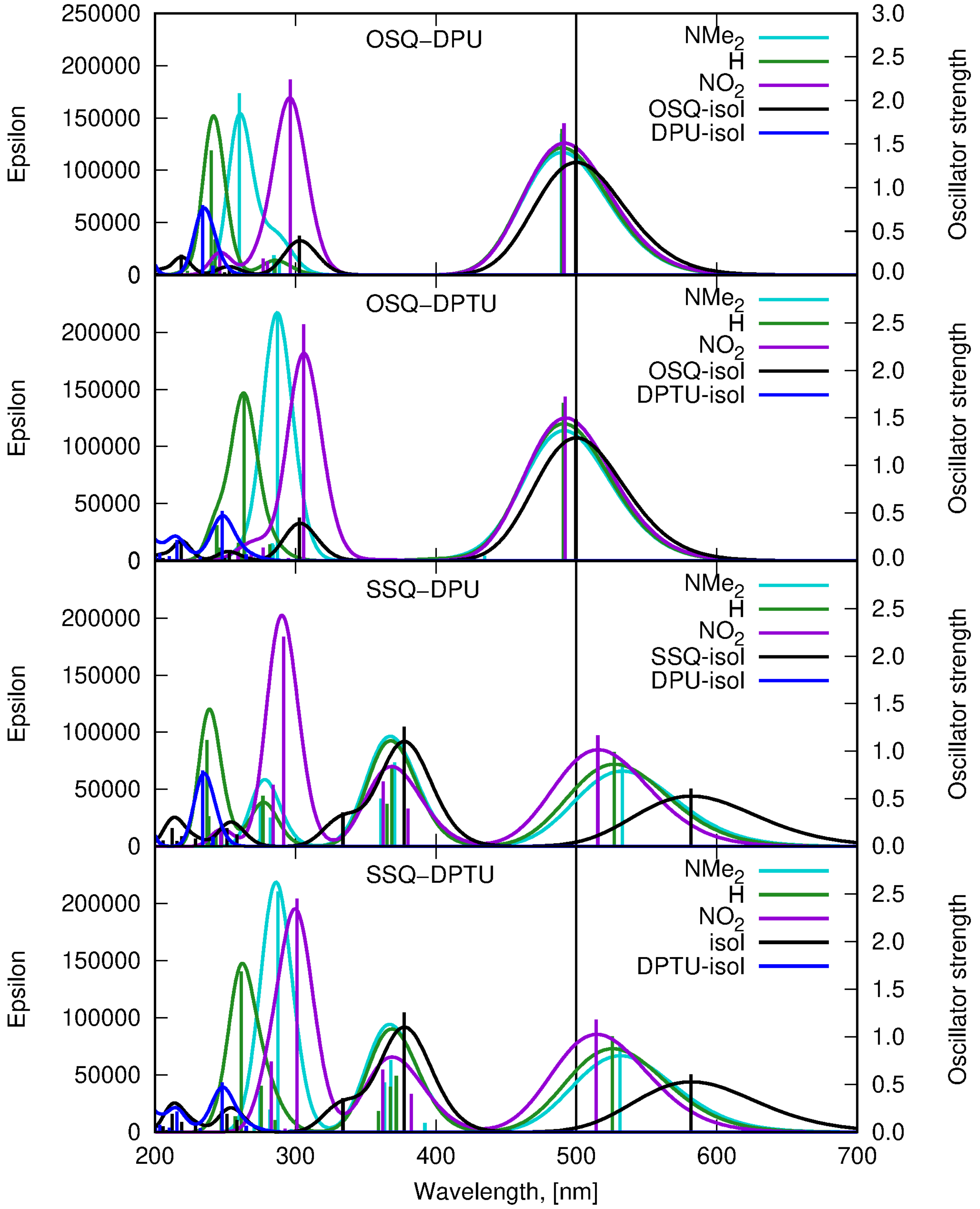

Figure 2: One-photon absorption spectrum for (thio)squaraine-N,N'-diphenyl(thio)urea complexes estimated within the CAM-B3LYP/6-31+G(d) approach in comparison to the isolated (thio)squaraine spectrum (black curve; vertical black line at 500 nm is added for the easier data comparison) and isolated N,N'-diphenyl(thio)urea (blue curve)

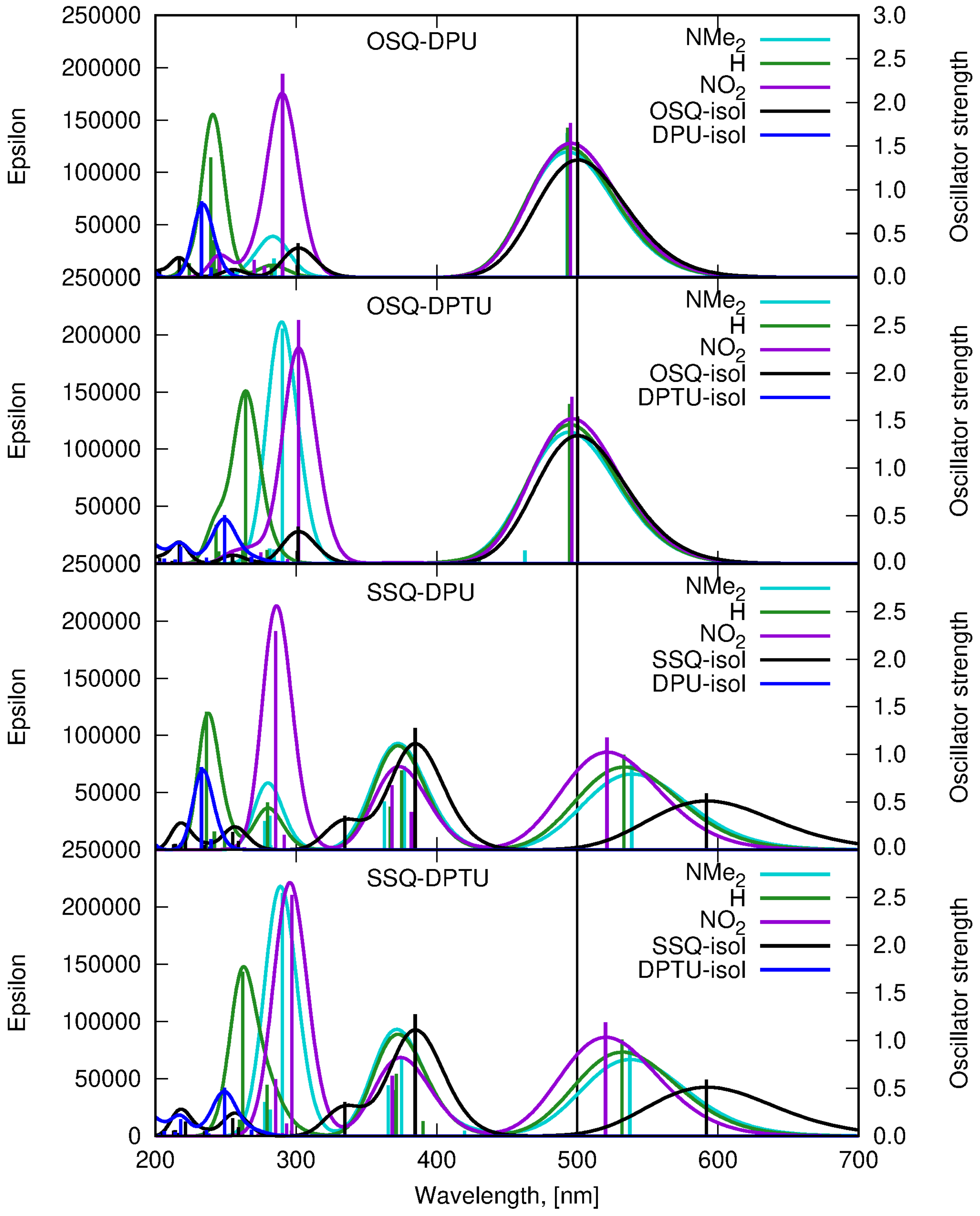

Figure 3: One-photon absorption spectrum estimated in vacuum within the M06-2X/6-31+G(d) approach (vertical black line at 500 nm is added for the easier data comparison)

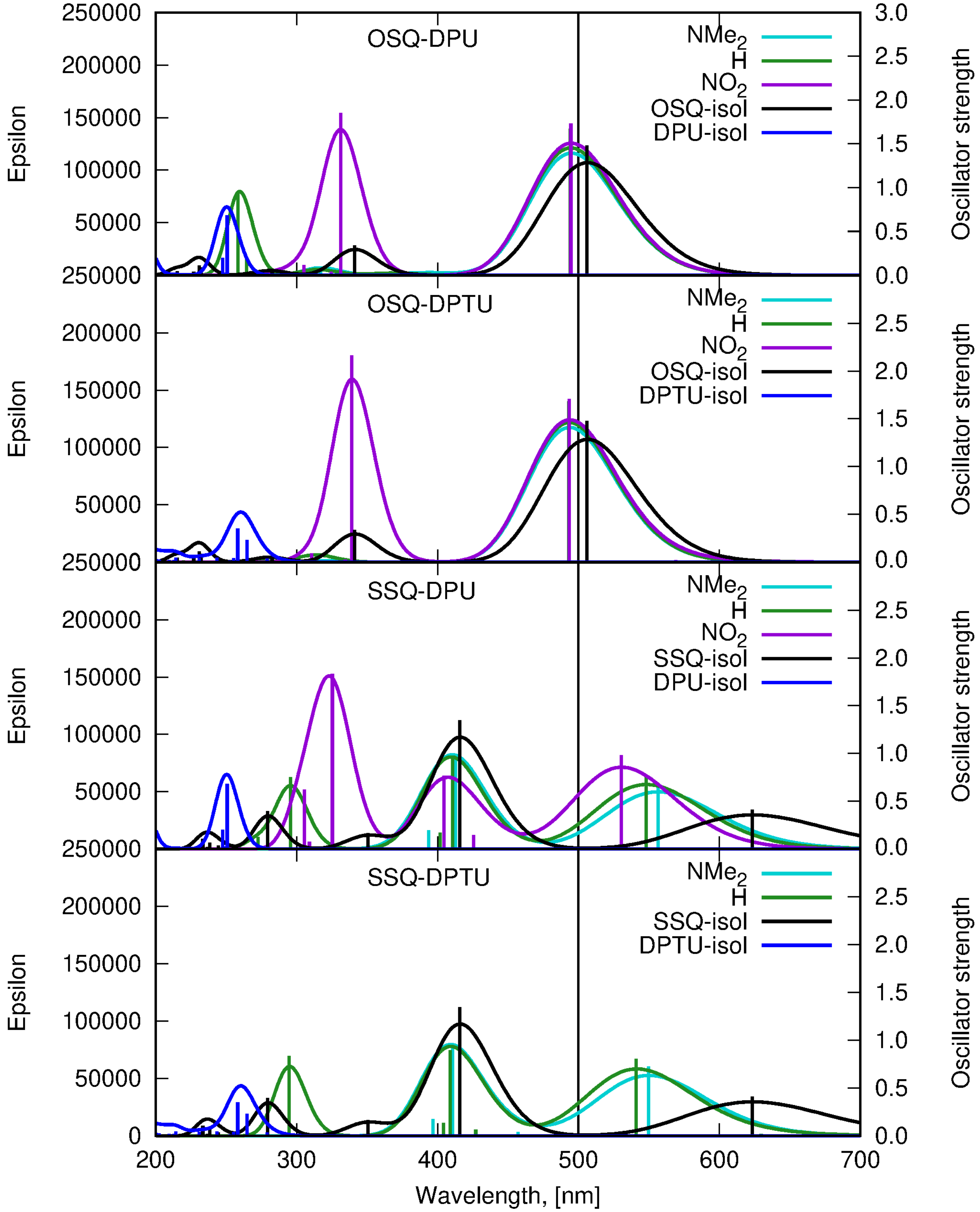

Figure 4: One-photon absorption spectrum estimated in vacuum within the PBE0/6-31+G(d) approach (vertical black line at 500 nm is added for the easier data comparison)

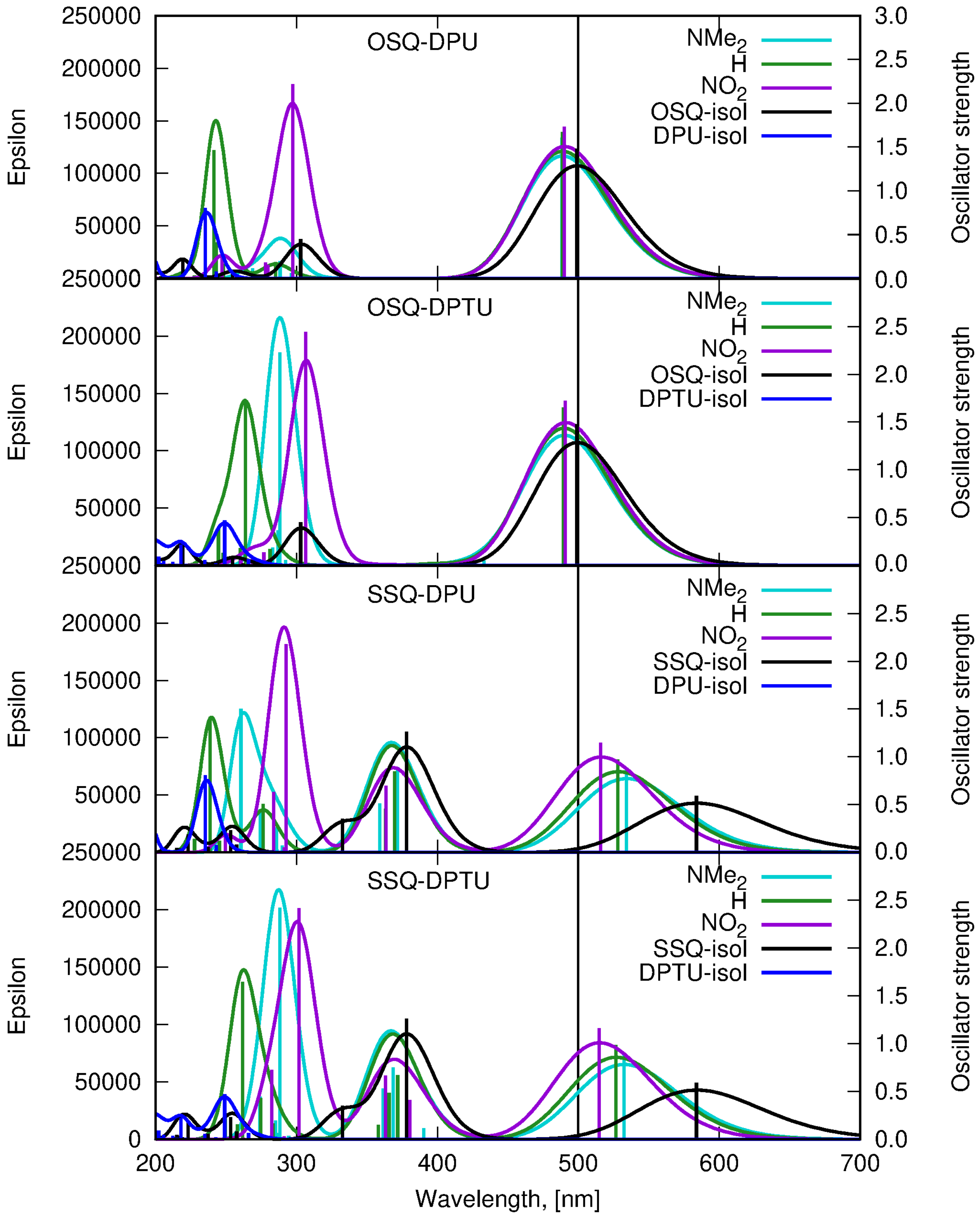

Figure 5: One-photon absorption spectrum estimated in vacuum within the CAM-B3LYP/6-311++G(d,p) approach (vertical black line at 500 nm is added for the easier data comparison)

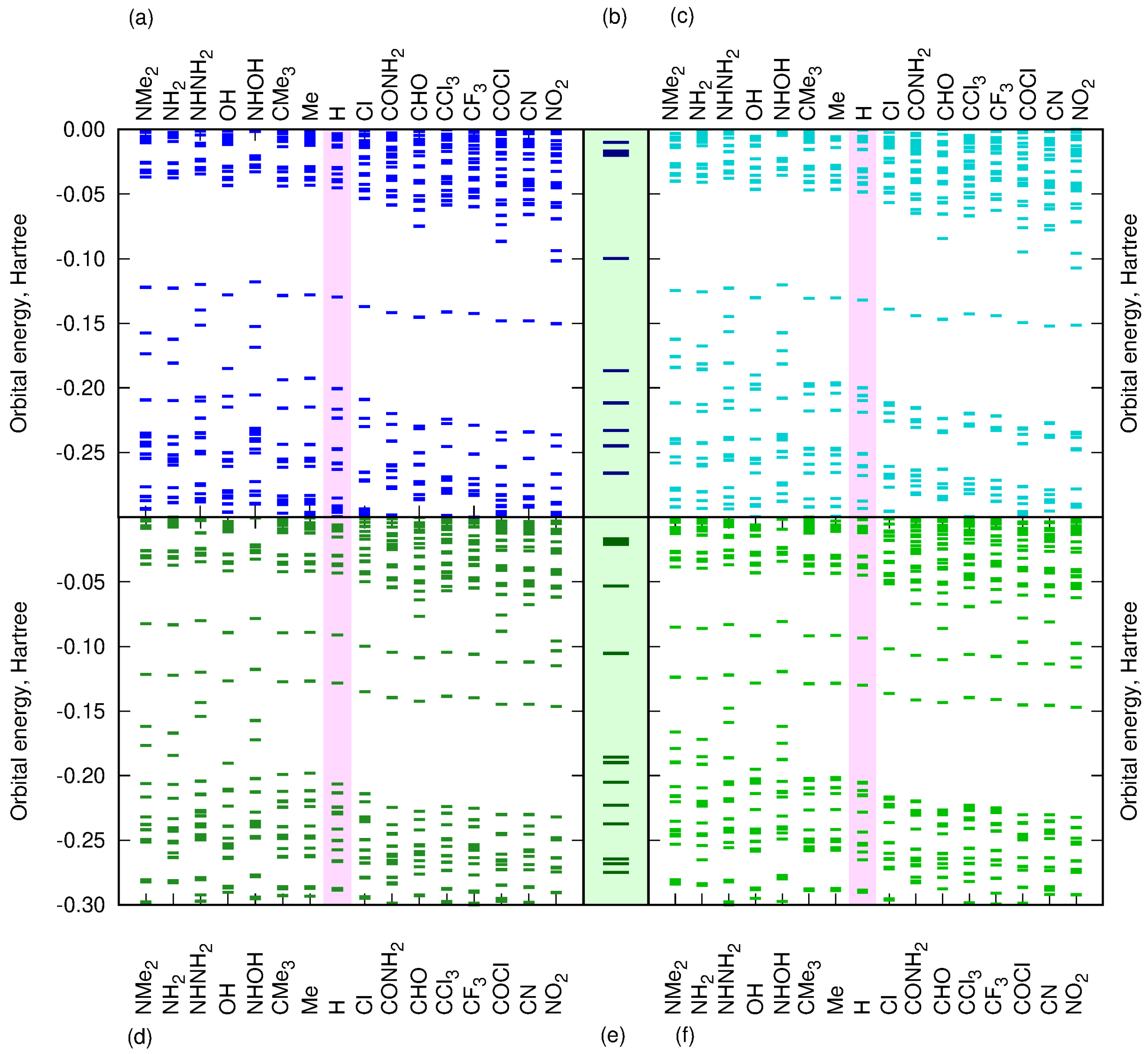

Figure 6: Orbital energy levels (B3LYP/6-311++G(d,p) together with the corresponding frontier orbitals for hydrogen-bonded squaraine and thiosquaraine complexes with two substituted N,N'-diphenyl(thio)urea molecules (magenta background emphasizes the unsubstituted reference complex)

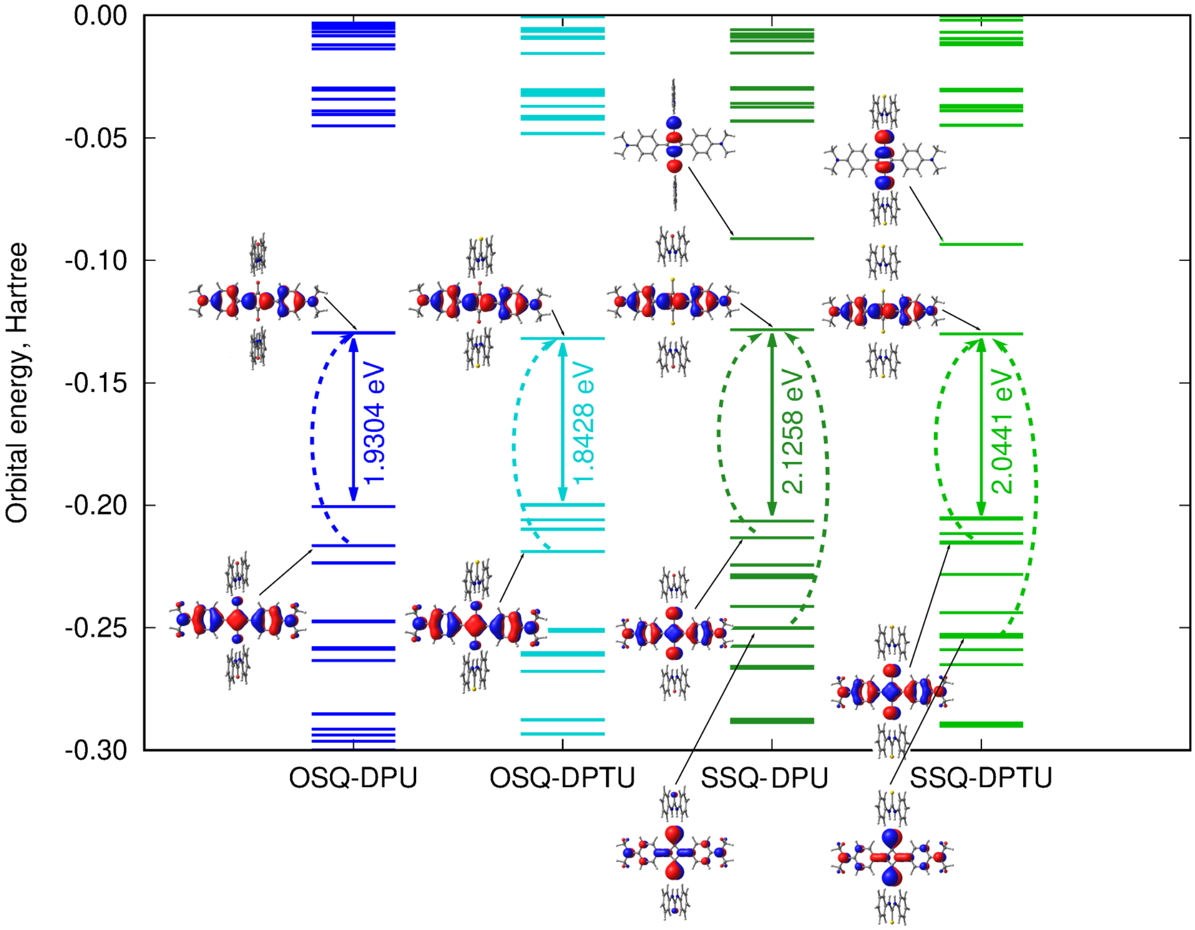

Figure 7: Orbital energy levels (B3LYP/6-311++G(d,p) together with the corresponding orbitals for hydrogen-bonded (thio)squaraine complexes with two N,N'-diphenyl(thio)urea molecules (the strait solid arrows indicate the HOMO–LUMO gap and arc dashed arrows connect the orbitals involved in the most intensive long-range transition)

Table 1: AIM parameters for the intermolecular hydrogen bonds in investigated systems (ρ denotes the electron density distribution, $\nabla^2\rho$ – its gradient and E_{HB} represents the intramolecular hydrogen bond energy in kcal/mol[1])

	OSQ-DPU			OSQ-DPTU			SSQ-DPU			SSQ-DPTU		
Substituent	ρ	$\nabla^2\rho$	E_{HB}	ρ	$\nabla^2\rho$	E_{HB}	ρ	$\nabla^2\rho$	E_{HB}	ρ	$\nabla^2\rho$	E_{HB}
$-NMe_2$	0.0215	0.0899	5.0778	0.0227	0.0944	5.4751	0.0099	0.0309	1.6197	0.0102	0.0313	1.6753
$-NH_2$	0.0214	0.0897	5.0689	0.0226	0.0943	5.4659	0.0099	0.0309	1.6217	0.0103	0.0315	1.6875
$-NHNH_2$	0.0212	0.0890	5.0079	0.0225	0.0938	5.4246	0.0098	0.0305	1.5921	0.0101	0.0310	1.6516
–OH	0.0222	0.0927	5.3057	0.0234	0.0971	5.7028	0.0105	0.0324	1.7158	0.0107	0.0328	1.7727
–NHOH	0.0214	0.0897	5.0672	0.0227	0.0945	5.4805	0.0100	0.0310	1.6239	0.0103	0.0315	1.6866
$-CMe_3$	0.0222	0.0926	5.3054	0.0235	0.0978	5.7632	0.0105	0.0324	1.7161	0.0107	0.0327	1.7680
–Me	0.0221	0.0924	5.2892	0.0234	0.0972	5.7118	0.0104	0.0322	1.7056	0.0106	0.0325	1.7545
–H	0.0224	0.0934	5.3642	0.0236	0.0979	5.7773	0.0106	0.0327	1.7343	0.0107	0.0328	1.7718
–Cl	0.0229	0.0956	5.5558	0.0241	0.1000	5.9623	0.0112	0.0344	1.8525	0.0113	0.0345	1.8881
$-CONH_2$	0.0230	0.0959	5.5864	0.0242	0.1003	5.9897	0.0112	0.0343	1.8477	0.0111	0.0339	1.8483
–CHO	0.0237	0.0983	5.7893	0.0247	0.1021	6.1607	0.0116	0.0356	1.9369	0.0116	0.0353	1.9463
$-CCl_3$	0.0235	0.0978	5.7434	0.0247	0.1021	6.1608	0.0116	0.0356	1.9375	0.0118	0.0357	1.9740
$-CF_3$	0.0234	0.0974	5.7164	0.0247	0.1020	6.1462	0.0116	0.0356	1.9367	0.0118	0.0356	1.9712
–COCl	0.0241	0.0998	5.9308	0.0254	0.1046	6.3951	0.0120	0.0367	2.0185	0.0121	0.0366	2.0449
–CN	0.0238	0.0986	5.8245	0.0323								
$-NO_2$	0.0242	0.1003	5.9787	0.0254	0.1045	6.3933	0.0121	0.0369	2.0370	0.0122	0.0369	2.0633

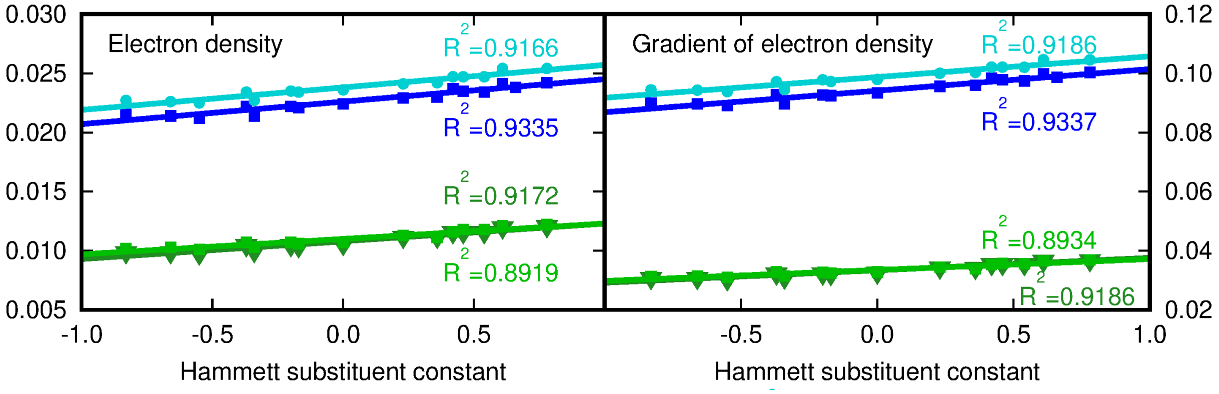

Figure 8: The correlation between the AIM electron density and its gradient and the Hammett substituent constant in the investigated complexes

Table 2: Le Bahers' charge transfer index $\Delta\sigma^2$ for the analyzed complexes (CAM-B3LYP/6-31G+G(d))

System	σ_p	sqO-DPU	sqO-DPTU	sqS-DPU	sqS-DPTU
$-NMe_2$	-0.83	-0.644	-0.742	-0.151	-0.226
–H	0.00	-0.651	-0.695	-0.228	-0.236
$-NO_2$	0.78	-0.695	-0.711	-0.301	-0.343

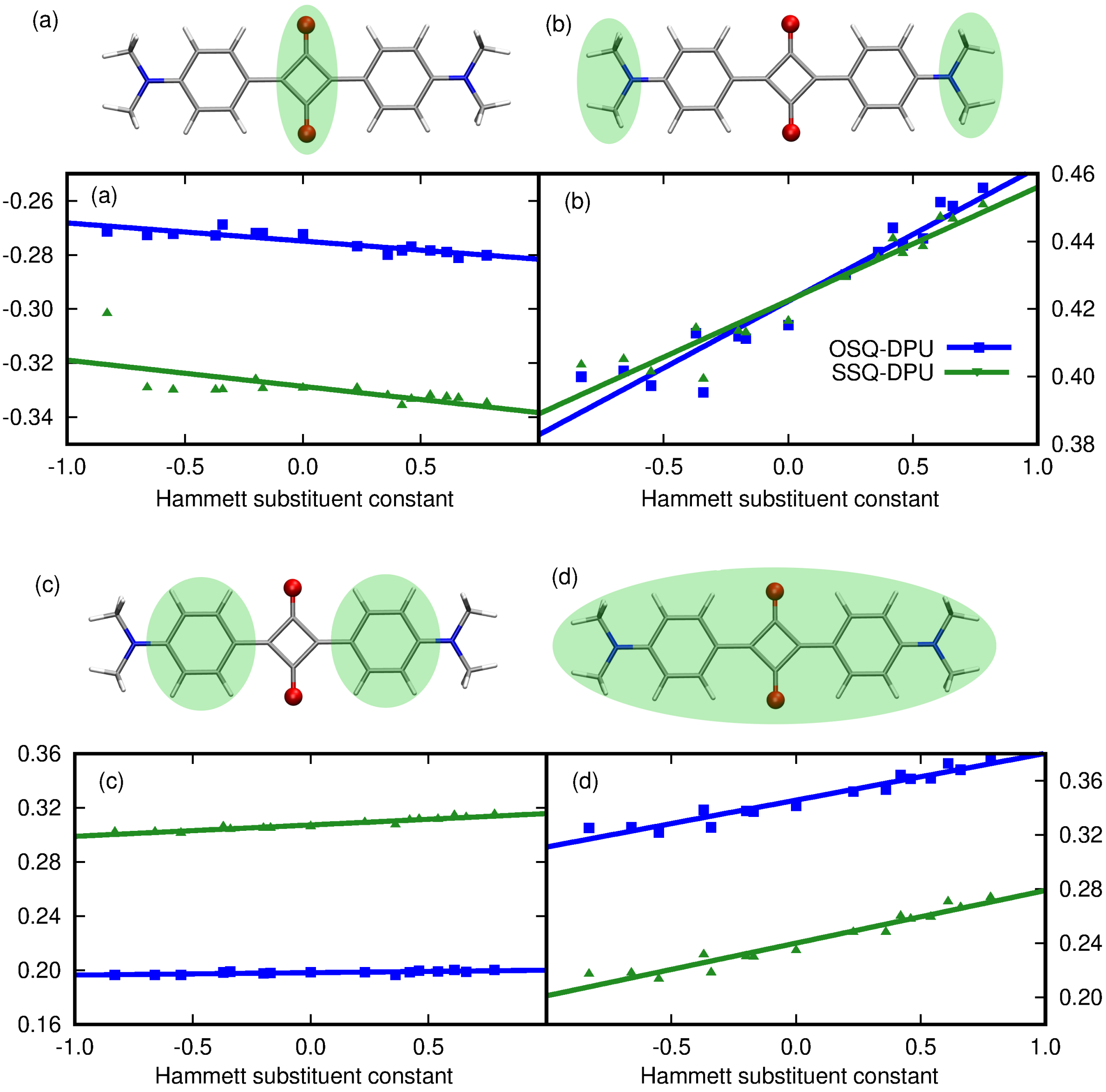

Figure 9: Modification of the Hirshfeld partial charge distribution in central (thio)squaraine unit upon substitution in N,N'-diphenylurea fragments: (a) in the central squaric ring, (b) in the terminal N,N-dimethylamino groups, (c) in phenyl rings, (d) in the whole (thio)squaraine moiety. Sum of partial charges from the considered fragment, presented in the green background, is given as an ordinate.

Table 3: AIM parameters for the ring critical point in the squaric ring in investigated systems (ρ denotes the electron density distribution, $\nabla^2\rho$ – its gradient)

	OSQ-DPU		OSQ-DPTU		SSQ-DPU		SSQ-DPTU	
Substituent	ρ	$\nabla^2\rho$	ρ	$\nabla^2\rho$	ρ	$\nabla^2\rho$	ρ	$\nabla^2\rho$
$-NMe_2$	0.0968	0.5016	0.0970	0.5020	0.1017	0.4874	0.1017	0.4873
$-NH_2$	0.0968	0.5016	0.0970	0.5020	0.1017	0.4874	0.1017	0.4873
$-NHNH_2$	0.0968	0.5015	0.0970	0.5019	0.1017	0.4874	0.1017	0.4873
–OH	0.0969	0.5019	0.0971	0.5023	0.1017	0.4874	0.1018	0.4874
–NHOH	0.0969	0.5017	0.0970	0.5022	0.1017	0.4875	0.1018	0.4875
$-CMe_3$	0.0969	0.5019	0.0971	0.5023	0.1017	0.4874	0.1018	0.4873
–Me	0.0969	0.5018	0.0971	0.5023	0.1017	0.4874	0.1018	0.4873
–H	0.0969	0.5019	0.0971	0.5023	0.1017	0.4874	0.1018	0.4873
–Cl	0.0970	0.5020	0.0972	0.5024	0.1017	0.4874	0.1018	0.4873
$-CONH_2$	0.0970	0.5019	0.0971	0.5022	0.1017	0.4872	0.1017	0.4871
–CHO	0.0971	0.5021	0.0972	0.5024	0.1017	0.4873	0.1018	0.4872
$-CCl_3$	0.0971	0.5022	0.0972	0.5025	0.1018	0.4873	0.1018	0.4873
$-CF_3$	0.0971	0.5021	0.0972	0.5025	0.1017	0.4873	0.1018	0.4872
–COCl	0.0971	0.5023	0.0973	0.5027	0.1018	0.4873	0.1018	0.4872
–CN	0.0971	0.5021	0.0975	0.5031				
$-NO_2$	0.0972	0.5023	0.0973	0.5026	0.1018	0.4872	0.1018	0.4872

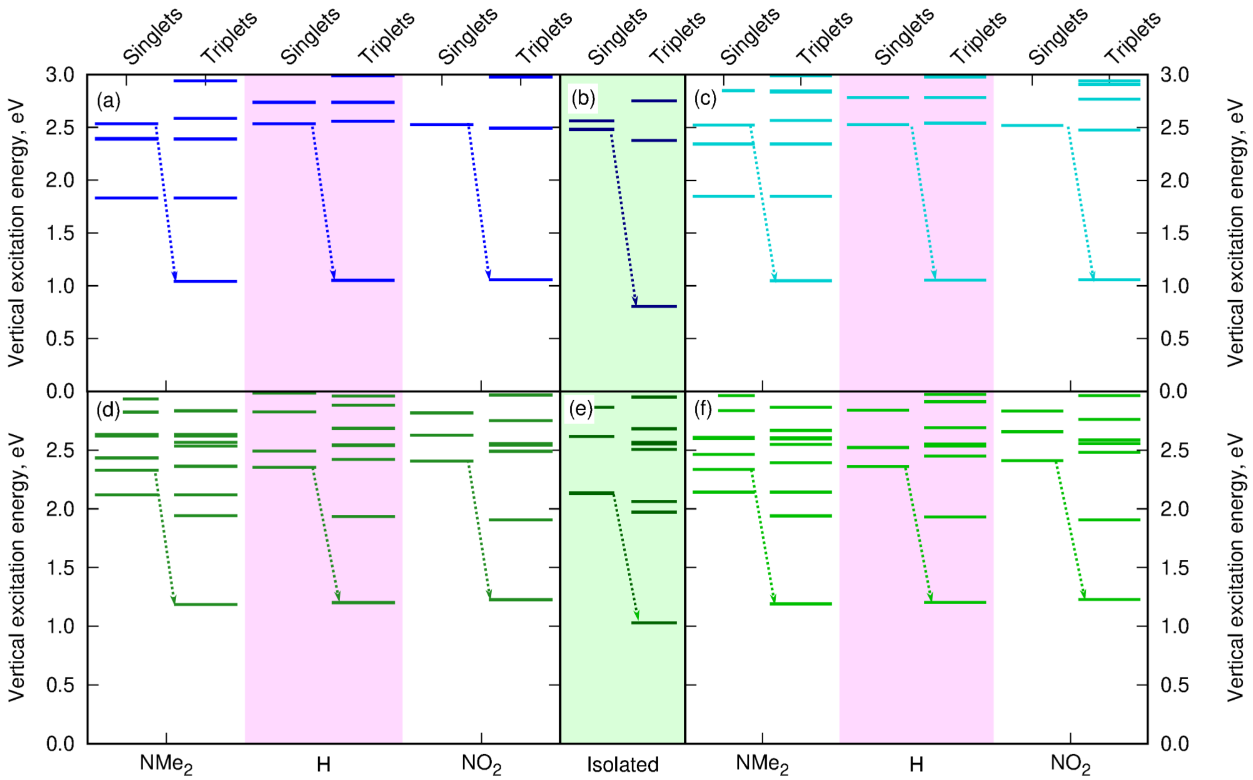

Figure 10: Singlet and triplet vertical excitation energy estimated within the CAM-B3LYP/6-31+G(d) approach for hydrogen-bonded (thio)squaraine complexes with two N,N'-diphenyl(thio)urea molecules (green background indicates the isolated (thio)squaraine energies and magenta background – the complexes with unsubstituted urea derivatives; the dottet arrows show the singlet-triplet energy gap for active states): (a) OSQ-DPU, (b) isolated OSQ, (c) OSQ-DPTU, (d) SSQ-DPU, (e) isolated SSQ, (f) SSQ-DPTU

Table 4: Supermolecular counterpoise-corrected interaction energy ωB97X-D/6-31+G(d) (the difference in the last row denotes the difference of the interaction energy between the most elecronodonating-substituted urea/thiourea and most electronoaccepting-substituted urea/thiourea complex)

System	σ_p	sqO-DPU	sqO-DPTU	sqS-DPU	sqS-DPTU
$-NMe_2$	-0.83	-28.93	-31.70	-13.68	-15.40
$-NH_2$	-0.66	-29.13	-31.94	-13.89	-15.66
$-NHNH_2$	-0.55	-28.33	-31.14	-13.33	-15.13
–OH	-0.37	-31.64	-34.46	-15.63	-17.43
–NHOH	-0.34	-28.48	-31.29	-13.21	-14.92
$-CMe_3$	-0.20	-31.57	-34.23	-15.41	-17.07
–Me	-0.17	-31.36	-34.02	-15.30	-16.95
–H	0.00	-32.22	-34.84	-15.89	-17.53
–Cl	0.23	-35.07	-37.74	-18.13	-19.90
$-CONH_2$	0.36	-35.47	-37.74	-18.43	-19.98
–CHO	0.42	-37.22	-39.52	-19.55	-21.07
$-CCl_3$	0.46	-36.92	-39.47	-19.33	-21.02
$-CF_3$	0.54	-37.08	-39.62	-19.53	-21.22
–COCl	0.61	-39.24	-41.55	-21.00	-22.53
–CN	0.66	-38.62	-39.61	-20.77	-22.43
$-NO_2$	0.78	-39.79	-42.10	-21.54	-23.15
Difference		-10.86	-10.41	-7.86	-7.75

Table 5: Supermolecular counterpoise-corrected interaction energy ωB97X-D/6-311++G(d,p) (the difference in the last row denotes the difference of the interaction energy between the most elecronodonating-substituted urea/thiourea and most electronoaccepting-substituted urea/thiourea complex)

System	σ_p	sqO-DPU	sqO-DPTU	sqS-DPU	sqS-DPTU
–NMe_2	-0.83	-29.48	-32.19	-14.00	-15.66
–NH_2	-0.66	-29.74	-32.50	-14.24	-15.95
–$NHNH_2$	-0.55	-28.94	-31.70	-13.68	-15.41
–OH	-0.37	-32.25	-35.01	-15.97	-17.70
–NHOH	-0.34	-29.10	-31.86	-13.57	-15.22
–CMe_3	-0.20	-32.16	-34.78	-15.76	-17.35
–Me	-0.17	-31.96	-34.57	-15.64	-17.22
–H	0.00	-32.86	-35.43	-16.25	-17.81
–Cl	0.23	-35.66	-38.27	-18.47	-20.17
–$CONH_2$	0.36	-36.09	-38.32	-18.79	-20.27
–CHO	0.42	-37.87	-40.13	-19.93	-21.38
–CCl_3	0.46	-37.54	-40.05	-19.69	-21.31
–CF_3	0.54	-37.83	-40.32	-19.98	-21.60
–COCl	0.61	-39.90	-42.18	-21.37	-22.84
–CN	0.66	-39.31	-40.52	-21.16	-22.76
–NO_2	0.78	-40.49	-42.75	-21.93	-23.48
Difference		11.01	10.56	7.93	7.82

Table 6: Supermolecular counterpoise-corrected interaction energy MP2/6-31+G(d) (the difference in the last row denotes the difference of the interaction energy between the most elecronodonating-substituted urea/thiourea and most electronoaccepting-substituted urea/thiourea complex)

System	σ_p	sqO-DPU	sqO-DPTU	sqS-DPU	sqS-DPTU
$–NMe_2$	-0.83	-27.62	-30.31	-14.01	-15.73
$–NH_2$	-0.66	-27.88	-30.61	-14.23	-15.99
$–NHNH_2$	-0.55	-27.16	-29.88	-13.72	-15.51
–OH	-0.37	-30.20	-32.96	-15.89	-17.68
–NHOH	-0.34	-27.17	-29.88	-13.50	-15.20
$–CMe_3$	-0.20	-30.12	-32.76	-15.78	-17.50
–Me	-0.17	-29.80	-32.42	-15.55	-17.24
–H	0.00	-30.57	-33.16	-16.11	-17.78
–Cl	0.23		-35.95	-18.25	-20.10
$–CONH_2$	0.36	-33.69	-36.03	-18.65	-20.36
–CHO	0.42	-35.19	-37.58	-19.59	-21.27
$–CCl_3$	0.46	-35.07	-37.68	-19.48	-21.31
$–CF_3$	0.54	-35.31	-37.86	-19.71	-21.51
–COCl	0.61	-37.17	-39.55	-21.03	-22.72
–CN	0.66	-36.54	-36.73	-20.75	-22.54
$–NO_2$	0.78	-37.41	-39.84	-21.35	-23.12
Difference		9.79	9.53	7.34	7.39

Table 7: One-photon maximum absorption wavelength [nm] calculated within the CAM-B3LYP/6-31+G(d) approach

System	σ_p	sqO-DPU	sqO-DPTU	sqS-DPU	sqS-DPTU
$–NMe_2$	-0.83	490	491	533	531
$–NH_2$	-0.66	490	491	532	530
$–NHNH_2$	-0.55	490	491	533	531
–OH	-0.37	490	491	527	525
–NHOH	-0.34	489	490	532	530
$–CMe_3$	-0.20	490	491	528	527
–Me	-0.17	490	491	528	527
–H	0.00	490	491	527	526
–Cl	0.23	490	491	522	520
$–CONH_2$	0.36	491	492	522	521
–CHO	0.42	491	492	519	519
$–CCl_3$	0.46	491	492	520	519
$–CF_3$	0.54	491	492	519	518
–COCl	0.61	491	492	516	516
–CN	0.66	491	492	517	516
$–NO_2$	0.78	491	492	515	514
Shift		1	1	18	17

Cartesian coordinates

OSQ-DPU

100

C	-0.000000	-0.000000	1.033834
C	-0.000000	0.000000	-1.033834
C	-0.000000	-0.000000	2.440693
C	-0.000000	0.000000	-2.440693
C	-0.000000	-0.000000	5.273195
C	-0.000000	0.000000	-5.273195
N	-0.000000	-0.000000	6.632531
N	-0.000000	0.000000	-6.632531
C	1.029659	-0.000000	-0.000000
C	-1.029659	-0.000000	0.000000
O	2.264808	-0.000000	-0.000000
O	-2.264808	-0.000000	0.000000
C	1.215637	0.000000	-3.166365
C	-1.215637	-0.000000	3.166365
C	1.215637	0.000000	3.166365
C	-1.215637	-0.000000	-3.166365
C	1.220161	0.000000	-4.540389
C	-1.220161	-0.000000	4.540389
C	1.220161	0.000000	4.540389
C	-1.220161	-0.000000	-4.540389
H	2.156024	0.000000	-2.622459
H	-2.156024	-0.000000	2.622459
H	2.156024	0.000000	2.622459
H	-2.156024	-0.000000	-2.622459
H	2.171271	0.000000	-5.058148
H	-2.171271	-0.000000	5.058148
H	2.171271	0.000000	5.058148
H	-2.171271	-0.000000	-5.058148
C	1.255047	0.000000	-7.366213
C	-1.255047	-0.000000	7.366213
C	1.255047	0.000000	7.366213
C	-1.255047	-0.000000	-7.366213
H	1.044100	0.000000	-8.435416
H	-1.044100	-0.000000	8.435416
H	1.044100	0.000000	8.435416
H	-1.044100	-0.000000	-8.435416
H	1.851673	0.891531	-7.138054
H	-1.851673	-0.891531	7.138054
H	1.851673	0.891531	7.138054
H	-1.851673	0.891531	7.138054
H	1.851673	-0.891531	7.138054
H	-1.851673	0.891531	-7.138054
H	1.851673	-0.891531	-7.138054
H	-1.851673	-0.891531	-7.138054
H	3.951494	-1.001637	-0.000000
H	-3.951494	-1.001637	0.000000
H	3.951494	1.001637	-0.000000
H	-3.951494	1.001637	-0.000000
N	4.956891	-1.140780	-0.000000
N	-4.956891	-1.140780	0.000000

N	4.956891	1.140780	-0.000000
N	-4.956891	1.140780	-0.000000
C	5.735623	-0.000000	-0.000000
C	-5.735623	-0.000000	0.000000
O	6.958744	-0.000000	-0.000000
O	-6.958744	-0.000000	0.000000
C	5.375744	-2.478330	-0.000000
C	-5.375744	-2.478330	0.000000
C	5.375744	2.478330	-0.000000
C	-5.375744	2.478330	-0.000000
C	4.366514	-3.453535	-0.000000
C	-4.366514	-3.453535	0.000000
C	4.366514	3.453535	-0.000000
C	-4.366514	3.453535	-0.000000
C	4.687466	-4.804943	-0.000000
C	-4.687466	-4.804943	0.000000
C	4.687466	4.804943	-0.000000
C	-4.687466	4.804943	-0.000000
C	6.020772	-5.212642	-0.000000
C	-6.020772	-5.212642	0.000000
C	6.020772	5.212642	-0.000000
C	-6.020772	5.212642	-0.000000
C	7.020625	-4.243661	-0.000000
C	-7.020625	-4.243661	0.000000
C	7.020625	4.243661	-0.000000
C	-7.020625	4.243661	-0.000000
C	6.716102	-2.883767	-0.000000
C	-6.716102	-2.883767	0.000000
C	6.716102	2.883767	-0.000000
C	-6.716102	2.883767	-0.000000
H	3.323121	-3.143989	-0.000000
H	-3.323121	-3.143989	0.000000
H	3.323121	3.143989	-0.000000
H	-3.323121	3.143989	-0.000000
H	3.888816	-5.541906	-0.000000
H	-3.888816	-5.541906	0.000000
H	3.888816	5.541906	-0.000000
H	-3.888816	5.541906	-0.000000
H	6.274293	-6.268606	-0.000000
H	-6.274293	-6.268606	0.000000
H	6.274293	6.268606	-0.000000
H	-6.274293	6.268606	-0.000000
H	8.065222	-4.543561	-0.000000
H	-8.065222	-4.543561	0.000000
H	8.065222	4.543561	-0.000000
H	-8.065222	4.543561	-0.000000
H	7.500132	-2.139028	-0.000000
H	-7.500132	-2.139028	0.000000
H	7.500132	2.139028	-0.000000
H	-7.500132	2.139028	-0.000000

OSQ-DPTU

100

C	-0.000000	0.000000	1.034516
C	-0.000000	0.000000	-1.034516
C	-0.000000	0.000000	2.440769
C	-0.000000	0.000000	-2.440769
C	-0.000000	0.000000	5.273371
C	-0.000000	0.000000	-5.273371
N	-0.000000	0.000000	6.632025
N	-0.000000	0.000000	-6.632025
C	1.028300	0.000000	-0.000000
C	-1.028300	0.000000	0.000000
O	2.264055	0.000000	-0.000000
O	-2.264055	0.000000	0.000000
C	1.215809	-0.000000	-3.166735
C	-1.215809	-0.000000	3.166735
C	1.215809	0.000000	3.166735
C	-1.215809	-0.000000	-3.166735
C	1.220441	-0.000000	-4.540395
C	-1.220441	-0.000000	4.540395
C	1.220441	0.000000	4.540395
C	-1.220441	-0.000000	-4.540395
H	2.156403	-0.000000	-2.623315
H	-2.156403	-0.000000	2.623315
H	2.156403	0.000000	2.623315
H	-2.156403	-0.000000	-2.623315
H	2.171547	-0.000000	-5.058089
H	-2.171547	-0.000000	5.058089
H	2.171547	0.000000	5.058089
H	-2.171547	-0.000000	-5.058089
C	1.255181	-0.000000	-7.366037
C	-1.255181	-0.000000	7.366037
C	1.255181	0.000000	7.366037
C	-1.255181	-0.000000	-7.366037
H	1.043997	-0.000000	-8.435142
H	-1.043997	-0.000000	8.435142
H	1.043997	0.000000	8.435142
H	-1.043997	-0.000000	-8.435142
H	1.851598	0.891578	-7.137873
H	-1.851598	-0.891578	7.137873
H	1.851598	0.891578	7.137873
H	-1.851598	0.891578	7.137873
H	1.851598	-0.891578	7.137873
H	-1.851598	0.891578	-7.137873
H	1.851598	-0.891578	-7.137873
H	-1.851598	-0.891578	-7.137873
H	3.967560	-0.930308	-0.000000
H	-3.967560	-0.930308	0.000000
H	3.967560	0.930308	-0.000000
H	-3.967560	0.930308	-0.000000
N	4.966052	-1.119865	-0.000000
N	-4.966052	-1.119865	0.000000
N	4.966052	1.119865	-0.000000
N	-4.966052	1.119865	-0.000000
C	5.757272	0.000000	-0.000000
C	-5.757272	0.000000	0.000000
S	7.429608	0.000000	-0.000000

S	-7.429608	0.000000	0.000000
C	5.229207	-2.502643	-0.000000
C	-5.229207	-2.502643	0.000000
C	5.229207	2.502643	-0.000000
C	-5.229207	2.502643	-0.000000
C	4.085127	-3.319773	-0.000000
C	-4.085127	-3.319773	0.000000
C	4.085127	3.319773	-0.000000
C	-4.085127	3.319773	-0.000000
C	4.195839	-4.702772	-0.000000
C	-4.195839	-4.702772	0.000000
C	4.195839	4.702772	-0.000000
C	-4.195839	4.702772	-0.000000
C	5.452656	-5.307128	-0.000000
C	-5.452656	-5.307128	0.000000
C	5.452656	5.307128	-0.000000
C	-5.452656	5.307128	-0.000000
C	6.584749	-4.499320	-0.000000
C	-6.584749	-4.499320	0.000000
C	6.584749	4.499320	-0.000000
C	-6.584749	4.499320	-0.000000
C	6.489972	-3.107851	-0.000000
C	-6.489972	-3.107851	0.000000
C	6.489972	3.107851	-0.000000
C	-6.489972	3.107851	-0.000000
H	3.099293	-2.858560	-0.000000
H	-3.099293	-2.858560	0.000000
H	3.099293	2.858560	-0.000000
H	-3.099293	2.858560	-0.000000
H	3.294277	-5.309258	-0.000000
H	-3.294277	-5.309258	0.000000
H	3.294277	5.309258	-0.000000
H	-3.294277	5.309258	-0.000000
H	5.544745	-6.389145	-0.000000
H	-5.544745	-6.389145	0.000000
H	5.544745	6.389145	-0.000000
H	-5.544745	6.389145	-0.000000
H	7.573068	-4.950906	-0.000000
H	-7.573068	-4.950906	0.000000
H	7.573068	4.950906	-0.000000
H	-7.573068	4.950906	-0.000000
H	7.382388	-2.499978	-0.000000
H	-7.382388	-2.499978	0.000000
H	7.382388	2.499978	-0.000000
H	-7.382388	2.499978	-0.000000

SSQ-DPU

100

C	-0.000000	0.000000	1.022477
C	-0.000000	0.000000	-1.022477
C	-0.000000	0.000000	2.431374
C	-0.000000	0.000000	-2.431374

C	-0.000000	0.000000	5.272763
C	-0.000000	0.000000	-5.272763
N	-0.000000	0.000000	6.630986
N	-0.000000	0.000000	-6.630986
C	1.033742	0.000000	-0.000000
C	-1.033742	0.000000	0.000000
S	2.692028	0.000000	-0.000000
S	-2.692028	0.000000	0.000000
C	1.214283	0.000000	-3.164597
C	-1.214283	0.000000	3.164597
C	1.214283	0.000000	3.164597
C	-1.214283	-0.000000	-3.164597
C	1.218241	0.000000	-4.537848
C	-1.218241	0.000000	4.537848
C	1.218241	0.000000	4.537848
C	-1.218241	-0.000000	-4.537848
H	2.156721	0.000000	-2.626833
H	-2.156721	0.000000	2.626833
H	2.156721	0.000000	2.626833
H	-2.156721	-0.000000	-2.626833
H	2.170567	0.000000	-5.053527
H	-2.170567	0.000000	5.053527
H	2.170567	0.000000	5.053527
H	-2.170567	-0.000000	-5.053527
C	1.255173	0.000000	-7.365039
C	-1.255173	0.000000	7.365039
C	1.255173	0.000000	7.365039
C	-1.255173	-0.000000	-7.365039
H	1.044054	0.000000	-8.434201
H	-1.044054	0.000000	8.434201
H	1.044054	0.000000	8.434201
H	-1.044054	-0.000000	-8.434201
H	1.851568	0.891486	-7.136621
H	-1.851568	-0.891486	7.136621
H	1.851568	0.891486	7.136621
H	-1.851568	0.891486	7.136621
H	1.851568	-0.891486	7.136621
H	-1.851568	0.891486	-7.136621
H	1.851568	-0.891486	-7.136621
H	-1.851568	-0.891486	-7.136621
H	5.050944	-1.033035	-0.000000
H	-5.050944	-1.033035	0.000000
H	5.050944	1.033035	-0.000000
H	-5.050944	1.033035	-0.000000
N	6.056121	-1.145885	-0.000000
N	-6.056121	-1.145885	0.000000
N	6.056121	1.145885	-0.000000
N	-6.056121	1.145885	-0.000000
C	6.827699	0.000000	-0.000000
C	-6.827699	0.000000	0.000000
O	8.049279	0.000000	-0.000000
O	-8.049279	0.000000	0.000000
C	6.482724	-2.485139	-0.000000
C	-6.482724	-2.485139	0.000000
C	6.482724	2.485139	-0.000000
C	-6.482724	2.485139	-0.000000

C	5.477768	-3.463272	-0.000000
C	-5.477768	-3.463272	0.000000
C	5.477768	3.463272	-0.000000
C	-5.477768	3.463272	-0.000000
C	5.806753	-4.812751	-0.000000
C	-5.806753	-4.812751	0.000000
C	5.806753	4.812751	-0.000000
C	-5.806753	4.812751	-0.000000
C	7.142330	-5.212504	-0.000000
C	-7.142330	-5.212504	0.000000
C	7.142330	5.212504	-0.000000
C	-7.142330	5.212504	-0.000000
C	8.137136	-4.238719	-0.000000
C	-8.137136	-4.238719	0.000000
C	8.137136	4.238719	-0.000000
C	-8.137136	4.238719	-0.000000
C	7.825115	-2.880407	-0.000000
C	-7.825115	-2.880407	0.000000
C	7.825115	2.880407	-0.000000
C	-7.825115	2.880407	-0.000000
H	4.432185	-3.160480	-0.000000
H	-4.432185	-3.160480	0.000000
H	4.432185	3.160480	-0.000000
H	-4.432185	3.160480	-0.000000
H	5.012230	-5.553925	-0.000000
H	-5.012230	-5.553925	0.000000
H	5.012230	5.553925	-0.000000
H	-5.012230	5.553925	-0.000000
H	7.401721	-6.267004	-0.000000
H	-7.401721	-6.267004	0.000000
H	7.401721	6.267004	-0.000000
H	-7.401721	6.267004	-0.000000
H	9.183264	-4.532800	-0.000000
H	-9.183264	-4.532800	0.000000
H	9.183264	4.532800	-0.000000
H	-9.183264	4.532800	-0.000000
H	8.605247	-2.131879	-0.000000
H	-8.605247	-2.131879	0.000000
H	8.605247	2.131879	-0.000000
H	-8.605247	2.131879	-0.000000

SSQ-DPTU

100

C	0.000000	1.023002	0.000000
C	-0.000000	-1.023002	0.000000
C	0.000000	2.431524	0.000000
C	-0.000000	-2.431524	0.000000
C	0.000000	5.273085	0.000000
C	-0.000000	-5.273085	0.000000
N	0.000000	6.630870	0.000000
N	-0.000000	-6.630870	0.000000
C	1.033047	-0.000000	-0.000000

C	-1.033047	0.000000	0.000000
S	2.691337	-0.000000	-0.000000
S	-2.691337	0.000000	0.000000
C	1.214378	-3.164965	0.000000
C	-1.214378	3.164965	0.000000
C	1.214378	3.164965	0.000000
C	-1.214378	-3.164965	-0.000000
C	1.218406	-4.538003	0.000000
C	-1.218406	4.538003	0.000000
C	1.218406	4.538003	0.000000
C	-1.218406	-4.538003	-0.000000
H	2.156936	-2.627511	0.000000
H	-2.156936	2.627511	0.000000
H	2.156936	2.627511	0.000000
H	-2.156936	-2.627511	-0.000000
H	2.170718	-5.053661	0.000000
H	-2.170718	5.053661	0.000000
H	2.170718	5.053661	0.000000
H	-2.170718	-5.053661	-0.000000
C	1.255236	-7.365183	0.000000
C	-1.255236	7.365183	0.000000
C	1.255236	7.365183	0.000000
C	-1.255236	-7.365183	-0.000000
H	1.043899	-8.434267	0.000000
H	-1.043899	8.434267	0.000000
H	1.043899	8.434267	0.000000
H	-1.043899	-8.434267	-0.000000
H	1.851492	-7.136807	0.891528
H	-1.851492	7.136807	-0.891528
H	1.851492	7.136807	0.891528
H	-1.851492	7.136807	0.891528
H	1.851492	7.136807	-0.891528
H	-1.851492	-7.136807	0.891528
H	1.851492	-7.136807	-0.891528
H	-1.851492	-7.136807	-0.891528
H	5.082578	-0.000000	-0.950897
H	-5.082578	0.000000	-0.950897
H	5.082578	-0.000000	0.950897
H	-5.082578	-0.000000	0.950897
N	6.080853	-0.000000	-1.123905
N	-6.080853	0.000000	-1.123905
N	6.080853	-0.000000	1.123905
N	-6.080853	-0.000000	1.123905
C	6.867148	-0.000000	-0.000000
C	-6.867148	0.000000	0.000000
S	8.536937	-0.000000	-0.000000
S	-8.536937	0.000000	0.000000
C	6.347269	-0.000000	-2.510082
C	-6.347269	0.000000	-2.510082
C	6.347269	-0.000000	2.510082
C	-6.347269	-0.000000	2.510082
C	5.206324	-0.000000	-3.329781
C	-5.206324	0.000000	-3.329781
C	5.206324	-0.000000	3.329781
C	-5.206324	-0.000000	3.329781
C	5.324103	-0.000000	-4.712258

C	-5.324103	0.000000	-4.712258
C	5.324103	-0.000000	4.712258
C	-5.324103	-0.000000	4.712258
C	6.583602	-0.000000	-5.310629
C	-6.583602	0.000000	-5.310629
C	6.583602	-0.000000	5.310629
C	-6.583602	-0.000000	5.310629
C	7.712212	-0.000000	-4.498405
C	-7.712212	0.000000	-4.498405
C	7.712212	-0.000000	4.498405
C	-7.712212	-0.000000	4.498405
C	7.610944	-0.000000	-3.107376
C	-7.610944	0.000000	-3.107376
C	7.610944	-0.000000	3.107376
C	-7.610944	-0.000000	3.107376
H	4.216874	-0.000000	-2.875548
H	-4.216874	0.000000	-2.875548
H	4.216874	-0.000000	2.875548
H	-4.216874	-0.000000	2.875548
H	4.425156	-0.000000	-5.322299
H	-4.425156	0.000000	-5.322299
H	4.425156	-0.000000	5.322299
H	-4.425156	-0.000000	5.322299
H	6.680478	-0.000000	-6.392196
H	-6.680478	0.000000	-6.392196
H	6.680478	-0.000000	6.392196
H	-6.680478	-0.000000	6.392196
H	8.702566	-0.000000	-4.945259
H	-8.702566	0.000000	-4.945259
H	8.702566	-0.000000	4.945259
H	-8.702566	-0.000000	4.945259
H	8.500761	-0.000000	-2.496168
H	-8.500761	0.000000	-2.496168
H	8.500761	-0.000000	2.496168
H	-8.500761	-0.000000	2.496168

References

(1) Espinosa, E.; Mollins, E.; Lecomte, C. Hydrogen bond strengths revealed by topological analyses of experimentally observed electron densities. *Chem. Phys. Lett.* **1998**, *285*, 170–173.

(2) Bahers, T. L.; Adamo, C.; Ciofini, I. A Qualitative Index of Spatial Extent in Charge-Transfer Excitations. *J. Chem. Theory Comput.* **2011**, *7*, 2498–2506.
